# Proteomic Analysis of Mouse Cerebral Cortex Following Experimental Ischemic Stroke: Identifying Novel Biomarkers of Damage and Repair

**DOI:** 10.1007/s10571-025-01645-y

**Published:** 2025-12-04

**Authors:** Dominik Hamer, Ana Butorac, Daniela Petrinec, Monika Berecki, Vera M. Mendes, Bruno Manadas, Vanja Kelava, Branimir K. Hackenberger, Anton Glasnović, Marija Lovrić, Srećko Gajović, Marina Dobrivojević Radmilović

**Affiliations:** 1 Department of Histology and Embryology, BIMIS – Biomedical Research Center Šalata, Croatian Institute for Brain Research, University of Zagreb School of Medicine, Šalata 3, 10000 Zagreb, Croatia; 2 BICRO BIOCentre Ltd, 10000 Zagreb, Croatia; 3 Selvita Ltd, 10000 Zagreb, Croatia; 4 CNC - Center for Neuroscience and Cell Biology - UC Biotech - Parque Tecnológico de Cantanhede, 3060-197 Cantanhede, Portugal; 5 Department of Biology, Josip Juraj Strossmayer University of Osijek, 31000 Osijek, Croatia

**Keywords:** Mouse cerebral cortex, Ischemic injury, Middle cerebral artery occlusion, Magnetic resonance imaging, Proteomic analysis, Gene ontology

## Abstract

Stroke remains a major global health challenge due to its high mortality and significant socioeconomic burden. Despite advances in clinical management, effective diagnostic tools and therapeutic strategies remain limited. This study aimed to identify and expand the repertoire of biomarkers of damage and repair that could serve as potential diagnostic and prognostic tools across post-stroke phases. Twenty-three male wild-type mice were assigned according to three longitudinal time points to control pre-stroke, 24-hour acute, and 35-day chronic post-stroke groups. Ischemic injury was induced via a 30-minute middle cerebral artery occlusion Koizumi method. Magnetic resonance imaging and neurological scoring were used to assess lesion size and functional deficit acutely, as well as structural and functional recovery during the chronic phase. Proteomic profiling of the ipsilateral and contralateral cortices was performed using data-independent acquisition (DIA)-based MS method. Statistical analysis revealed 74 differentially expressed proteins showing significant temporal changes in expression, which were classified into four temporal expression clusters: *acutely and chronically upregulated*,* acutely upregulated and chronically downregulated*, *acutely downregulated and chronically upregulated*, and *acutely and chronically downregulated*. Gene ontology analysis identified 47 affected biological processes, including synaptic signaling, immune response, cell-cell communication, cytoskeletal organization, and proliferation. Thirteen proteins previously not associated with stroke pathophysiology were identified, including 10 from the ipsilateral cortex (*Dbi*, *Cpne3*, *Dnm2*, *Eef1a1*, *Taldo1*, *Pgls*, *Gnb5*, *Phf24*, *Ctsz*, *Capg*) and 3 from the contralateral cortex (*Agpat3*, *Cacng8*, *Endod*). The identified biomarkers provide novel molecular insights into post-stroke energy metabolism, neuroinflammation, and cellular remodeling, highlighting potential targets for further intervention.

**Graphical Abstract:**

Proteomic analysis of ipsilateral and contralateral mouse cortices post-stroke identified 13 previously unreported proteins, revealing distinct temporal expression patterns linked to tissue damage and repair. These findings highlight potential biomarkers with diagnostic, prognostic, or predictive value for ischemic stroke.

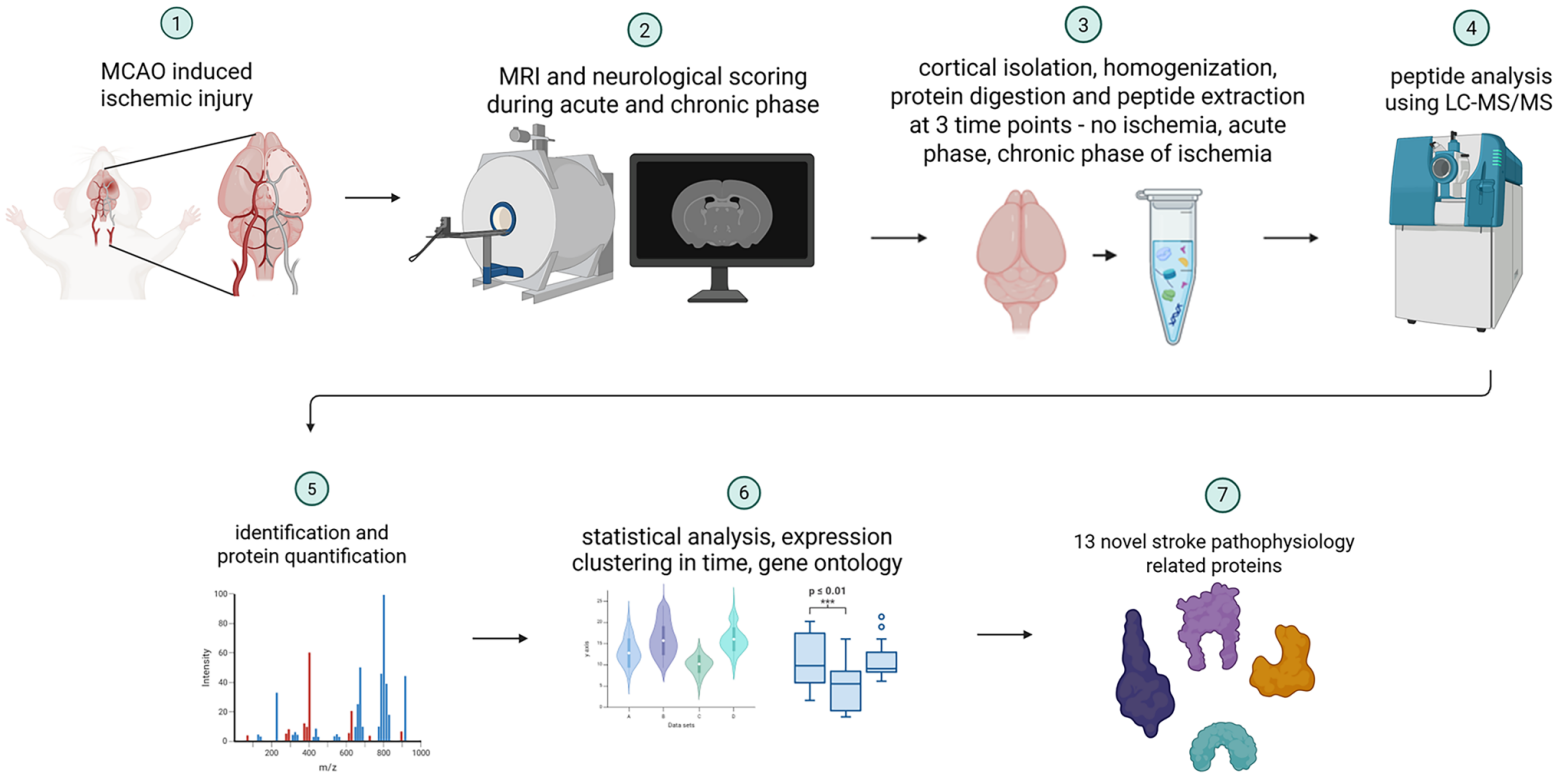

**Supplementary Information:**

The online version contains supplementary material available at 10.1007/s10571-025-01645-y.

## Introduction

Stroke is the second leading cause of death and the primary cause of long-term disability worldwide (Donnan et al. 2008; Barthels and Das 2020; Hilkens et al. 2024). Ischemic stroke, which arises from the occlusion of cerebral arteries due to thrombus formation, accounts for approximately 85% of all stroke cases. In contrast, hemorrhagic stroke results from the rupture of cerebral blood vessels, leading to bleeding into the surrounding brain tissue (Sirsat et al. 2020). Despite considerable progress in acute stroke care, therapeutic options for ischemic stroke remain largely confined to reperfusion strategies: either intravenous thrombolysis using recombinant tissue plasminogen activator (rt-PA) or endovascular thrombectomy (Donnan et al. 2008; Kristoffersen et al. 2025). Current diagnostic protocols rely heavily on neuroimaging techniques, which are indispensable for distinguishing stroke subtypes and assessing lesion extent. However, these imaging modalities offer limited insight into the biological processes underlying stroke evolution, prognosis, and the dynamics of neurological recovery. These limitations instigate the scientific community to explore molecular biomarkers that can serve as complementary tools for diagnosis, prognostication, and therapeutic monitoring. The identification of robust biomarkers reflecting post-ischemic injury and subsequent endogenous repair mechanisms has potential to significantly enhance clinical decision-making and patient stratification. Notably, no molecular biomarker has yet been validated for routine clinical use in ischemic stroke diagnosis and patient monitoring, emphasizing the critical need for further research in this area (Jickling and Sharp 2015; Dagonnier et al. 2021; Huang et al. 2023; Rahmig et al. 2024).

Preclinical stroke studies using animal models remain indispensable for understanding the pathophysiology of stroke and the mechanisms of damage and repair. Among these, the middle cerebral artery occlusion (MCAO) method in rodents is widely recognized for its ability to replicate key features of human ischemic stroke (Fluri et al. 2015; Matur et al. 2023). Ischemic brain injury triggers a cascade of molecular and cellular events such as oxidative stress, excitotoxicity, neuroinflammation, blood-brain barrier disruption, and neuronal death (Kaufmann et al. 1999; Uzdensky 2019; Maida et al. 2024). These processes are mediated by a complex network of proteins, many of which have been proposed as potential biomarkers (Sobsey et al. 2020). Candidate molecules include natriuretic peptides, albumin, glial fibrillary acidic protein (GFAP), neuron-specific enolase (NSE), interleukin-6 (IL-6), D-dimer, matrix metalloproteinases (MMPs), tumor necrosis factor-α (TNF-α), and interleukin-1 (IL-1) (Jickling and Sharp 2015; Wen et al. 2019; Gu et al. 2021; Gunton et al. 2017; Pawluk et al. 2022). Proteomics has emerged as a powerful and unbiased approach for identifying novel molecular markers associated with ischemic stroke. However, the majority of current research focuses on the acute phase of injury, with relatively little attention given to the molecular landscape of the chronic recovery phase (Datta et al. 2011; Chen et al. 2015; Li et al. 2020; Zheng et al. 2020; Agarwal et al. 2020). Using preclinical in vivo imaging in our previous studies, we have shown that the chronic phase following ischemia reveals important aspects of functional and structural recovery (Gorup et al. 2019; Romić et al. 2024). Moreover, we were able to reframe and distinguish the two most commonly used MCAO models, redefining the Koizumi method as a model of ischemia with chronic hypoperfusion, simulating spontaneous recanalization, and the Longa method as a model of ischemia with immediate reperfusion closely mimicking mechanical thrombectomy in humans (Justić et al. 2022). Building on these findings, the present study investigates changes in the cerebral cortical proteome across distinct phases of ischemic stroke in a mouse model. Specifically, we conducted a quantitative proteomic analysis at three critical time points: pre-lesion baseline, one day post-ischemia (acute phase), and 35 days post-ischemia (chronic phase). This approach enabled a comprehensive characterization of molecular alterations associated with both injury progression and recovery. By examining proteomic profiling in the context of temporal ischemic damage evolution, this study aims to identify novel molecular candidates with diagnostic, prognostic, or predictive potential for ischemic stroke.

## Materials and Methods

### Experimental Animals

All experimental procedures, including animal handling and euthanasia, were approved by the Ethics Committee and Animal Welfare Committee of the University of Zagreb School of Medicine (approval number 380-59-10106-23-111/25) and the Ministry of Agriculture of the Republic of Croatia (approval number UP/I-322-01/23 − 01/4). All researchers who conduct experimental procedures on animals are licensed to work with laboratory animals. The study followed the ARRIVE guidelines (Percie du Sert et al., 2020). Adult male mice were used, belonging to the albino congenic inbred strain (C57BL/6-Tyr^c−Brd/J^, 12–16 weeks old) bred at the animal facility at the Croatian Institute for Brain Research, University of Zagreb School of Medicine. The animals were housed under standard laboratory conditions, including a temperature-controlled environment at 22 ± 2 °C, a humidity-controlled environment, a 12-hour light/dark cycle, and *ad libitum* access to food and water. Animals were divided into three groups: control group (BL, baseline animals that did not undergo MCAO procedure; *n* = 5), acute phase MCAO group (D01; animals subjected to MCAO and euthanized 24 h after ischemic lesion; *n* = 5), chronic phase MCAO group (D35; animals subjected to MCAO and euthanized 35 days after ischemic lesion; *n* = 5). Sham-operated controls were not included in this study. To adhere to the 3Rs principle and minimize animal use, pre-stroke baseline animals were used as controls to specifically assess ischemia-related proteomic changes. In total, 23 animals were included in the study. Three animals were excluded due to intracerebral hemorrhage confirmed by magnetic resonance imaging (MRI), 4 animals did not survive to the study endpoint (D35), and brain tissue from one animal was allocated for proteomic method development. This resulted in a final cohort of 15 animals included in the proteomic analysis. All animals underwent baseline MRI 7 days prior to MCAO. Animals subjected to MCAO were additionally scanned on day 1 and day 35 post-surgery to confirm the presence and monitor the progression of ischemic injury. MRI data were complemented with neurological deficit assessments conducted at baseline (7 days before MCAO), and on days 1, 2, 3 and 35 post-MCAO. At the designated time points (BL, D01, D35), mice were euthanized, followed by transcardial perfusion, brain extraction, and ipsilateral and contralateral cortical dissection to obtain tissue for subsequent proteomic analysis. To reduce potential subjective bias, animals were randomly assigned to experimental groups using the lottery box method prior to any procedures. All investigators involved in conducting experimental interventions, including magnetic resonance imaging, neurological assessments, tissue harvesting, and proteomics, were blinded to the group allocation throughout the study. Furthermore, the investigators responsible for data processing and statistical analyses remained blinded to group identities until all analyses were completed to ensure unbiased interpretation of results.

### Transient Middle Cerebral Artery Occlusion (MCAO)

Ischemic brain injury was induced using the modified 30-minute Koizumi MCAO method using a silicon-coated monofilament (Koizumi et al. 1986; Justić et al. 2022). Mice were anesthetized with 4% isoflurane (Isofluran-Piramal, Piramal Critical Care, Germany) for induction and maintained at 1.5–2% isoflurane throughout the surgery. Before the surgery and following until 2 days after surgery, animals were administered intraperitoneally 0.250 mL of buprenorphine (0.05 mg/kg; Buprenovet, Bayer, Germany) for analgesia and 0.9% saline solution (0.5 mL) for rehydration. The body temperature was continuously monitored using a rectal temperature probe and maintained at 37 ± 0.5 °C with a temperature-controlled heating pad. To prevent corneal drying, the eyes were covered with ophthalmic ointment (Recugel, Bausch & Lomb, Canada). MCA blood flow was monitored using a laser-Doppler perfusion monitor (moorVMS-LDF1, Moor Instruments, UK) with a 1 mm diameter laser probe placed against the temporal bone. Temporal dynamics of the middle cerebral artery blood flow during the MCAO surgical procedure are provided in the Supplementary File 1 (Supplementary Fig. 1). After recording baseline blood flow values, the animals were positioned in a supine position, and the surgical field was shaved and disinfected. A midline neck incision was made to expose the left common carotid artery (CCA), internal carotid artery (ICA), and external carotid artery (ECA). Following permanent ligation of the CCA, a silicon-coated monofilament (Doccol Corporation, USA) was inserted into the CCA incision and advanced through the circle of Willis to the MCA origin. The monofilament remained in place for 30 min, after which the animals were re-anesthetized for filament removal, allowing reperfusion. After surgery, animals were placed in cages on a 37 °C heating pad for 24 h, with water and softened pelleted food provided to the animals in the Petri dishes to facilitate feeding and water intake.

### In Vivo Mouse Brain MRI, MRI Data Processing, Segmentation and Volumetric Brain Analysis

MRI was performed using a 7 T BioSpec 70/20 USR system (Bruker Biospin, Germany) equipped with Paravision 6.0.1 software in a transmit/receive (Tx/Rx) configuration. Signal transmission was achieved using an 86 mm transmit volume coil (MT0381, Bruker Biospin, Germany), while signal reception was performed with a two-element mouse brain surface receive coil (MT0042, Bruker Biospin, Germany). Mice were initially anesthetized using 4% isoflurane (Isofluran-Piramal, Piramal Critical Care, Germany) in a 30%/70% O_2_/N_2_ mixture. During MRI scanning, anesthesia was maintained at 1.5–2% isoflurane in the same gas mixture. Breathing rate was continuously monitored using an optical probe (medres, Cologne, Germany) and maintained at 80–100 breaths per minute. The body temperature was regulated at 37 ± 0.5 °C using a feedback-controlled circulating heating pump. Temperature monitoring was performed using an MR-compatible rectal temperature probe (medres, Cologne, Germany). The animals were positioned inside a water-heated Bruker mouse bed (Bruker, Germany), with the head stabilized using a tooth bar and ear bars to minimize motion artifacts. The total duration of the MRI procedure, including anesthesia induction, positioning, and scanning, was approximately 40 min. Imaging parameters and geometric settings for in vivo MRI of the mouse brain are provided in Supplementary File 1 (Supplementary Table 1). Mice were imaged 7 days before middle cerebral artery occlusion (MCAO; baseline imaging), 1-day post-MCAO (D01), and 35-day post-MCAO (D35). MR images were converted to NIfTI format using the Bru2Nii (GitHub Inc., San Francisco, SAD) tool to enable processing in ImageJ software version 1.53d (Wayne Rasband, National Institutes of Health, USA). Image segmentation was performed by manual delineation of the ipsilateral and contralateral hemispheres, as well as the ischemic lesion. Ischemic lesion was delineated on T2-map scans while brain hemispheres were segmented using T2-weighted scans. Additionally, ventricle volume was quantified from T2-map images using a semi-automatic macro protocol implemented in FIJI/ImageJ 1.53d software.

### Neurological Scoring and Weight Measurement

Neurological scoring (NS) and weight measurements were performed 7 days prior, on the days 1, 2, 3 and 35 after MCAO. The functional deficit of ischemia was assessed in several categories using composite neurological scale that adapts elements of the Garcia scale and the Bederson score, including appearance (0–12), spontaneous activity (0–5), gait (0–5), forelimb flexion (0–2), placing (0–4), thorax twisting (0–2), lateral resistance (0–2), and sensory function (0–7), yielding a maximum total score of 39 points, with higher scores indicating greater neurological deficit. Weight loss was assessed at the same time points (Bederson et al. 1986; Garcia et al. 1995; Justić et al. 2022, 2025). The tests were performed once, by the same person, before each MR imaging, 7 days before, and on days 1, 2, 3, and 35 after MCAO.

### Animal Euthanasia, Transcardial Perfusion and Tissue Isolation

Mice were anesthetized with intraperitoneal injection of 2.5% tribromoethanol (Avertin, Sigma Aldrich, USA) and transcardially perfused with 30 mL of phosphate-buffered saline (PBS, pH 7.4). After perfusion, brains were carefully extracted and transferred to a Petri dish filled with cold PBS. Each brain was then bisected into hemispheres, after which the entire cortex was dissected from the hemisphere by carefully separating the cortical mantle from the underlying subcortical structures along the white-matter boundary. After dissection, both ipsilateral and contralateral cortices were placed into 1.5 mL tubes, weighted, snap-frozen in liquid nitrogen, and stored at − 80 °C until further proteomic analysis.

### Proteomic Analysis

#### Cortical Tissue Processing and Protein Digestion

For sample preparation modified short-GeLC protocol (Anjo et al. 2017) was used. Based on the weighted cortical tissue mass, 1× Laemmli buffer (containing 15 mg/mL DTT) was added to achieve a final concentration of 200 mg of cortical tissue per mL of 1× Laemmli buffer. Homogenization was performed using ultrasound sonication (ultrasound amplitude: 40%; total duration of the homogenization per sample was 20 s in cycles: 3 s homogenization and 2 s break). Homogenized samples were denatured for 5 min at 95 °C. Acrylamide was added to a final concentration of 1% (v/v) to alkylate the reduced cysteines. The samples were centrifuged at 10,000 × g for 15 min at 4 °C. PierceTM 660 nm Protein Assay (Thermo Fisher Scientific, Illinois, USA) was used to measure the total protein concentration in the samples.

For comparative proteome analysis, individual sample volumes containing 50 µg of protein were loaded onto 4–20% Mini-PROTEAN^®^ TGX™ Precast Gels (Bio-Rad), with five replicates for each experimental condition. To facilitate protein identification and spectral library generation for ipsilateral and contralateral cortices, three pooled representative samples were prepared, designated as follows: P_B_ (pooled baseline samples), P_A_ (pooled acute phase (D01) samples), and P_C_ (pooled chronic phase (D35) samples). These pooled samples were prepared by combining equal amounts of protein from each biological replicate for their respective time points (BL, D01, and D35). To generate a spectral library, 70 µg of protein per time point was loaded onto 4–20% Mini-PROTEAN^®^ TGX™ Precast Gels (Bio-Rad). The proteins underwent partial electrophoretic separation, followed by in-gel digestion, according to the described protocol (Anjo et al. 2017). For in-gel protein digestion, 70 µL of a prepared trypsin (Promega, USA) solution (final concentration 0.01 µg/µL in 10 mM ammonium bicarbonate (Sigma-Aldrich, USA) prepared in ddH₂O) was added to each well. Samples were incubated at 4 °C for 15 min, after which an additional 70 µL of 10 mM ammonium bicarbonate solution was added to fully cover the gel pieces. Digestion was carried out overnight at room temperature. Excess solution from gel pieces was removed and transferred to a low-binding microcentrifuge tube (Thermo Fischer Scientific, USA). Three peptide extraction solutions were prepared with the following compositions: solution 1 (30% acetonitrile (Sigma-Aldrich, USA) and 1% formic acid (VWR, Radnor, Pennsylvania, USA), solution 2 (50% acetonitrile and 1% formic acid), and solution 3 (96% acetonitrile and 1% formic acid). Solution 1 (100 µL) was added to the gel pieces and incubated for 15 min at 25 °C using horizontal thermomixer (at 1200 rpm). Solution with extracted peptides was transferred to the same low-binding microcentrifuge tube referred above. The described steps for peptide extraction were repeated for solutions 2 and 3. The peptide-containing solution was evaporated using a vacuum concentrator (Eppendorf™ Concentrator Plus, Hamburg, Germany) until dry and stored in glass vials at − 80 °C for further LC − MS/MS analysis (Anjo et al. 2017).

#### LC − MS/MS Analysis

Peptides were separated and analyzed using a NanoLC™ 425 System (Eksigent Technologies, California, USA) coupled to a Triple TOF™ 6600 mass spectrometer (Sciex, Massachusetts, USA) equipped with an ESI DuoSpray™ Source (Sciex, Massachusetts, USA). Due to technical constraints of the study design, ipsilateral and contralateral cortical samples were analyzed on two separate, although identical models of mass spectrometry instruments. As a result, direct quantitative comparisons between hemispheres were not conducted, and the analysis was restricted to within-hemisphere longitudinal comparisons for each cortex independently. Peptide samples were resuspended in 30 µL of the mobile phase (2% of acetonitrile and 0.1% of formic acid in HPLC-grade water (Sigma-Aldrich, USA), sonicated for 2 minutes in a cuphorn (40% amplitude and 1 s “on” and 1 s “off” cycles) and centrifuged at 14,000 × g for 5 minutes. The supernatant was transferred to the appropriate LC vials, and 10 µL (16 µg) from a 30 µL sample in mobile phase was injected into a chromatographic column. Chromatographic separation was performed using a Triart C18 Capillary Column 1/32” (12 nm, S-3µm, 150 × 0.3 mm, YMC) with a corresponding Triart C18 Capillary Guard Column (0.5 × 5 mm, 3 µm, 12nm, YMC). The column was maintained at 50 °C, with a flow rate of 5 µL/min. Mobile phase A consisted of 5% DMSO plus 0.1% formic acid in HPLC-grade water, and mobile phase B was 5% DMSO plus 0.1% formic acid in acetonitrile. The LC gradient elution was programmed as follows: 5–30% of B (0–50 min), 30–98% of B (50–52 min), 98% of B (52–54 min), 98–5% of B (54–56 min), and 5% of B (56–65 min). To monitor instrument performance and ensure data consistency, a peptide standard mix (digested β-galactosidase) was injected before and after each sample run. The mass spectrometer was operated in the positive ion mode with the following ion source parameters: an ion spray voltage of 5500 V, nebulizer gas 1 (GS1) at 25 psi, nebulizer gas 2 (GS2) at 10 psi, curtain gas (CUR) at 25 psi, and source temperature (TEM) of 100°C. For protein identification and spectral library creation, a Data-dependent acquisition (DDA) method was used. The mass spectrometer was set to scan full spectra (m/z 350 − 1500) for 250 ms, followed by up to 100 MS/MS scans (m/z 100–2000). Ions selected for fragmentation were those with a charge state between +1 and +5 and counts exceeding the threshold of 100 counts per second. Each ion was analyzed once and then added to an exclusion list for 15 seconds. The rolling collision was used with a collision energy spread of 5. For quantitative proteome analysis, the sequential window acquisition of all theoretical mass spectra (SWATH-MS) data-independent acquisition (DIA) method was employed. The mass spectrometer was operated in a looped product ion mode and specifically tuned to cover 168 overlapping windows across the precursor mass range of m/z 350 − 2250. At the beginning of each cycle, a 50-ms survey scan covering the m/z range of 350 − 2250 was acquired. SWATH-MS/MS spectra were collected from m/z 100 − 2250 for 19 ms, resulting in an acquisition cycle time of 3.3 seconds. The mass spectrometer was operated using Analyst^®^ TF 1.8.1, Sciex^®^ (Sciex, Massachusetts, USA). The mass spectrometry proteomics data have been deposited to the ProteomeXchange Consortium via the PRIDE (Perez-Riverol et al. 2025) partner repository with the dataset identifier PXD067155.

#### MS Data Processing

Two distinct ion libraries, comprising precursor and fragment ions were independently created for the ipsilateral and contralateral hemispheres. The files from the pooled samples were combined into separate protein identification search using the ProteinPilot™ software (v5.0, Sciex). The searches were conducted with the Paragon method and included the following parameters: reviewed Mus musculus database from SwissProt (downloaded on February 2nd of 2023), cysteine alkylation by acrylamide, digestion by trypsin, and gel-based ID. To assess the quality of identification, an independent False Discovery Rate (FDR) analysis was performed using the target-decoy approach provided by Protein Pilot™. Quantitative data processing was performed using the SWATH processing plug-in for PeakView (v2.0.01, Sciex^®^). The quantitative analysis was based on the information obtained from the protein identification search. Peptides with less than 1% of FDR and a sum of up to 5 fragments/peptide were used for quantification. Data normalization was done for each protein based on the total sum of areas for the respective sample.

#### Relative Protein Quantification

For quantitative analysis of the protein results, two different approaches were employed. First, a non-parametric Kruskal–Wallis test was performed to select the proteins that were statistically different between the three conditions using the MetaboAnalyst web-based platform (Pang et al. 2020) as well as false discovery rate (FDR) as a multiple comparison method. In parallel, as cross-validation, a permutation test with *post-hoc* Bonferroni correction was performed due to the small sample size. The final list of differentially expressed proteins (DEPs) was created by overlapping the proteins identified as significant by the Kruskal–Wallis test (*p* < 0.05), the FDR analysis (*p* < 0.05), and the permutation test (*p* < 0.05). Volcano plots were made for ipsilateral and contralateral cortical differentially expressed proteins in both acute and chronic post-stroke phases compared to control using VolcaNoseR web-based platform (Goedhart and Luijsterburg 2020).

#### Mfuzz Time Series Clustering

DEPs were clustered using the R package Mfuzz (Kumar and Futschik, 2007), which were derived by analyzing the expression profiles of the DEPs across the time points in our study (BL, D01, and D35) based on their fold change (FC) values in each time point.

#### Enrichment Analysis

To investigate the involvement of statistically up- or down-regulated proteins and their corresponding genes in different metabolic processes, gene set enrichment analysis (GSEA) was performed using a free online bioinformatics computational model, GO Enrichment Analysis powered by PANTHER 18.0. (Protein ANalysis THrough Evolutionary Relationships; https://www.pantherdb.org/) Classification System (Mi and Thomas 2009). After entering gene names, GO parameters were set for “biological process”, “Mus musculus” and analysis was performed.

### Statistical Analysis

Statistical analysis of brain volumetric data and neuroscoring was performed using GraphPad Prism 9. (GraphPad Software, USA). The Shapiro-Wilk test was used to assess the normality of data distribution. Based on the results of normality tests, datasets were then analyzed using nonparametric and parametric statistics. For the evaluation of ipsilateral and contralateral cortex volume, swelling, and tissue loss, a mixed model one-way analysis of variance (ANOVA) was used. *Post-hoc* comparison was performed using the Bonferroni multiple-comparison correction test. Statistical analysis of proteomic data was performed using MetaboAnalyst 6.0. web-based software (https://www.metaboanalyst.ca) for Kruskal-Wallis and FDR test, and RStudio 4.4.1. for the permutation test. A *p* < 0.05 was considered statistically significant for all tests. Data were presented as median and interquartile range. For data visualization, GraphPadPrism, MetaboAnalyst, SRplot (https://www.bioinformatics.com.cn/srplot), Biorender (https://www.biorender.com/) and VolcaNoseR (https://huygens.science.uva.nl/VolcaNoseR/) were combined.

## Results

### Post-stroke Ischemic Brain Injury Extent and Animal Neurological Status

#### MR Imaging Data and Neurological Deficit Assessment

**Fig. 1:**
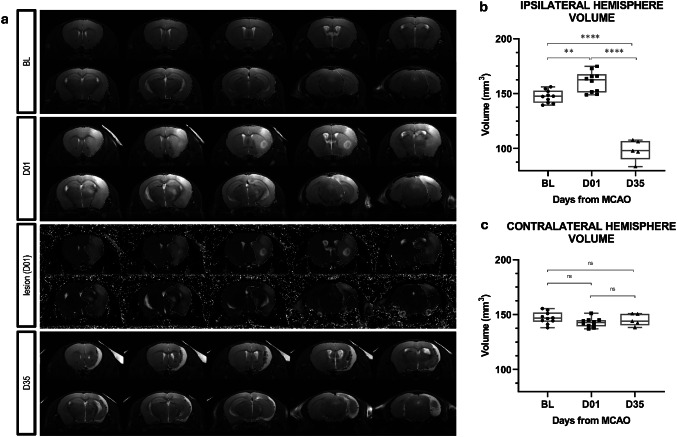
Temporal dynamics of hemisphere volume changes in MCAO-operated mice. (**a**) Representative high-resolution T2-weighted MR images showing in vivo hemisphere volume changes at three time points: baseline (BL), acute post-stroke phase (D01), and chronic post-stroke phase (D35). The hyperintense signal at D01 in T2-weighted and T2 map images indicates the presence of an ischemic lesion. Segmented hemispheres and lesions are outlined. By D35, the lesion site is marked by the formation of an astroglial scar and compensatory ventriculomegaly. Ipsilateral (**b**) and contralateral (**c**) hemisphere volume dynamics measured before (baseline - BL), 1. day (D01), and 35. day (D35) after MCAO. Statistical analysis was performed using one-way ANOVA followed by Bonferroni multiple comparisons correction. Data are presented as median with interquartile range (BL n = 10; D01 n = 10; D35 n = 5). **p < 0.001, ****p < 0.0001

To assess the extent of morphological changes and characterize the ischemic lesion in each animal, *in vivo* MR imaging was performed across all experimental groups. Volumetric analysis of the hemispheres conducted at baseline confirmed comparable brain hemisphere volumes among the included animals (ipsilateral: 151.95 ± 5.93 mm³; contralateral: 151.98 ± 5.21 mm³), providing individual reference points for subsequent assessments of post-ischemic changes. Neuroimaging revealed cortico-striatal lesions in five animals (3 animals in the acute post-stroke phase group and 2 in the chronic (D35) post-stroke phase group) and cortico-striatal-hippocampal lesions in the remaining 5 animals (2 animals from the D01 group and 3 animals from the D35 group). Manual volumetric segmentation of the MR images revealed the development of substantial vasogenic edema in the ipsilateral hemisphere during the acute phase (90.14 ± 22.60 mm³), occupying over 50% of the hemisphere volume (Fig. 1a). The ipsilateral edema induced a compensatory compression of the contralateral hemisphere, reflected by a transient reduction in its volume (147.68 ± 4.39 mm³), while the ipsilateral hemisphere expanded markedly (163.76 ± 9.54 mm³). In the chronic phase, the volume of the contralateral hemispheres returned to the initial values (151.73 ± 5.95 mm³), whereas the ipsilateral hemisphere exhibited significant tissue loss (104.73 ± 12.42 mm³) (Fig. 1b, c). Comprehensive volumetric data, including individual measurements of ipsilateral and contralateral hemisphere volume, lesion size, and tissue loss, are presented in the Supplementary File 2 (Tables 1 and 2).

In parallel, assessment of animal body weight and neurological status indicated a marked decline in overall animal condition and motor performance in the acute phase after stroke, with the most pronounced neurological deficits observed on day 2 post-MCAO. By day 35, animals demonstrated partial functional recovery, with neurological scores approaching baseline values. Detailed neurological scoring for each animal across five time points (BL, D01, D02, D03, and D35), including category-specific and total scores, is provided in Supplementary File 2 (Table 3).

### Stroke-Induced Changes in Cerebral Cortical Proteome

#### Temporal Profiling and Enrichment Analysis of Differentially Expressed Proteins in the Mouse Cortex

**Fig. 2:**
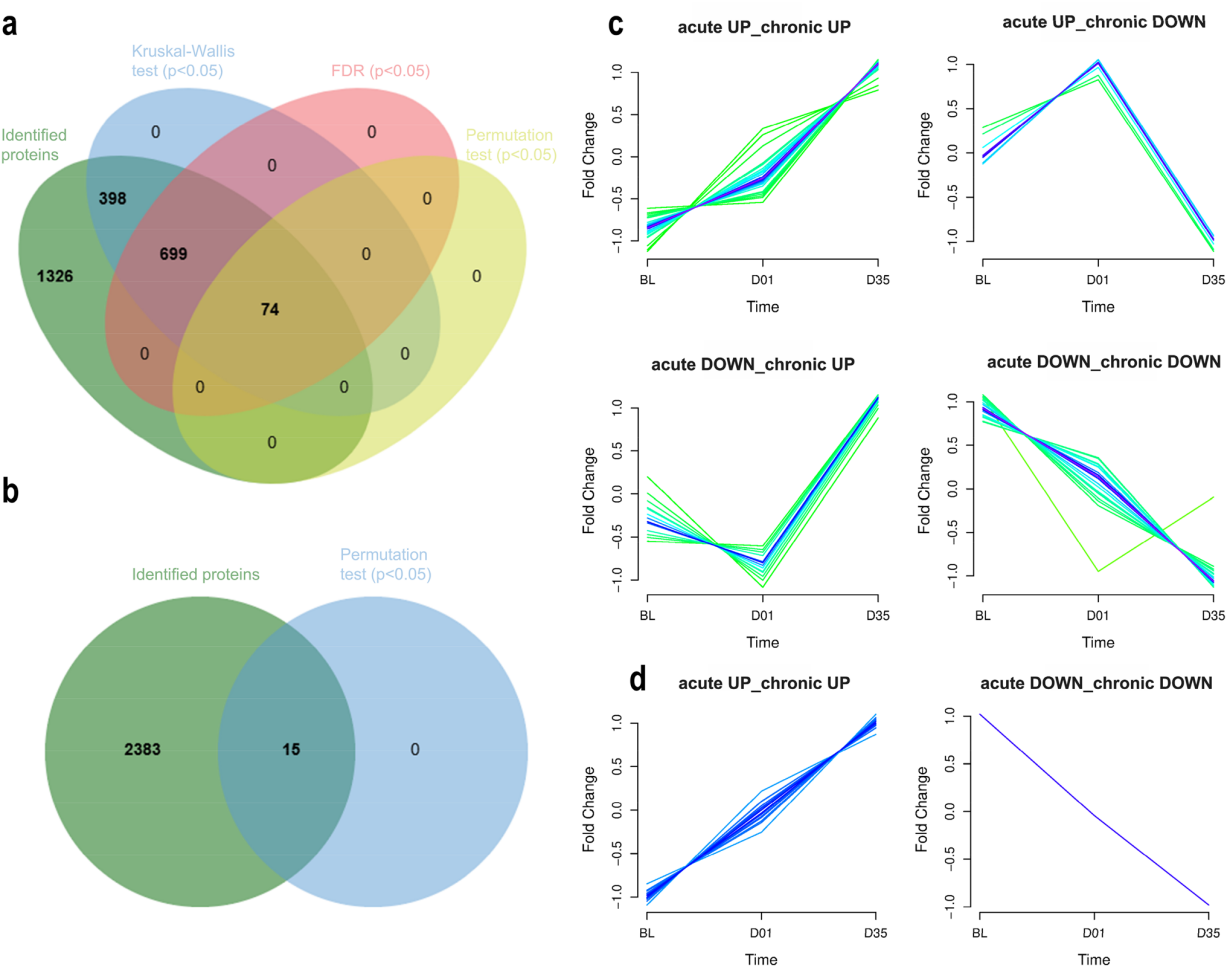
Proteomic changes and temporal clustering of stroke-responsive cortical proteins. Number of differentially expressed proteins (DEPs) associated with altered enriched biological processes based on Kruskal-Wallis analysis, false discovery rate, and permutation test with Bonferroni correction in the (**a**) ipsilateral (*N* = 74) and (**b**) contralateral (*N* = 15) mouse cerebral cortex following middle cerebral artery occlusion. (**c**,** d**) Temporal expression dynamics of differentially expressed proteins (DEPs) were analyzed using the MFuzz clustering algorithm for omics time-series data. (**c**) Ipsilateral DEPs were divided into four discrete clusters: acute upregulation with sustained upregulation in the chronic phase (*acute UP_chronic UP*; *N* = 29), acute upregulation followed by downregulation (*acute UP_chronic DOWN*; *N* = 8), acute downregulation with later upregulation (*acute DOWN_chronic UP*; *N* = 15) and sustained downregulation (*acute DOWN_chronic DOWN*; *N* = 22). (**d**) In the contralateral cortex, DEPs were divided into two temporal profiles: *acute UP_chronic UP* (*N* = 14) and *acute DOWN_chronic DOWN* (*N* = 1)

To investigate the molecular changes induced by ischemic injury in the MCAO model, we performed a quantitative proteomic analysis of cortical tissues. Ipsilateral and contralateral cortices were analyzed separately and compared to non-ischemic control animals. In the ipsilateral cortices, across the three experimental time points (BL, D01, and D35), a total of 2497 proteins were identified and quantified (normalized proteomic data available in Supplementary File 2; Table 4). Statistical analysis revealed 74 differentially expressed proteins (DEPs) showing significant temporal changes in expression across the three conditions. To identify these DEPs, we first applied the non-parametric Kruskal–Wallis test. This analysis identified 1171 proteins with statistically significant differences in expression across time points (*p* < 0.05). To control for multiple testing, we applied the false discovery rate correction, which reduced the number of significantly altered proteins to 773. To further refine our findings, we conducted a permutation-based test with *post-hoc* Bonferroni correction for multiple comparisons. This method, which provides robust statistical inference in small-sample proteomic studies, was based on 50,000 permutations and included pairwise comparisons between all experimental groups (BL vs. D01, BL vs. D35, and D01 vs. D35). This stringent approach identified 74 proteins with statistically significant changes in expression (Fig. 2a). A complete list of p-values from the permutation test is provided in the Supplementary File 1; Supplementary Table 2, and the full list of ipsilateral cortices DEPs is available in the Supplementary File 1; Supplementary Table 3.

Proteomic profiling of the contralateral cortex identified a total of 2398 proteins (normalized proteomic data in Supplementary File 2; Table 5). However, only a small subset of these proteins demonstrated statistically significant alterations over time. Specifically, the permutation test with *post-hoc* Bonferroni correction revealed 15 proteins with significant changes, while the Kruskal–Wallis test and FDR correction did not identify any significant differences, indicating a relatively modest proteomic response in the contralateral, non-ischemic hemisphere (Fig. 2b). A complete list of p-values from the permutation test is provided in the Supplementary File 1; Supplementary Table 2, while the complete list of these 15 significant DEPs of the contralateral cortex is available in Supplementary File 1; Supplementary Table 4.

**Fig. 3:**
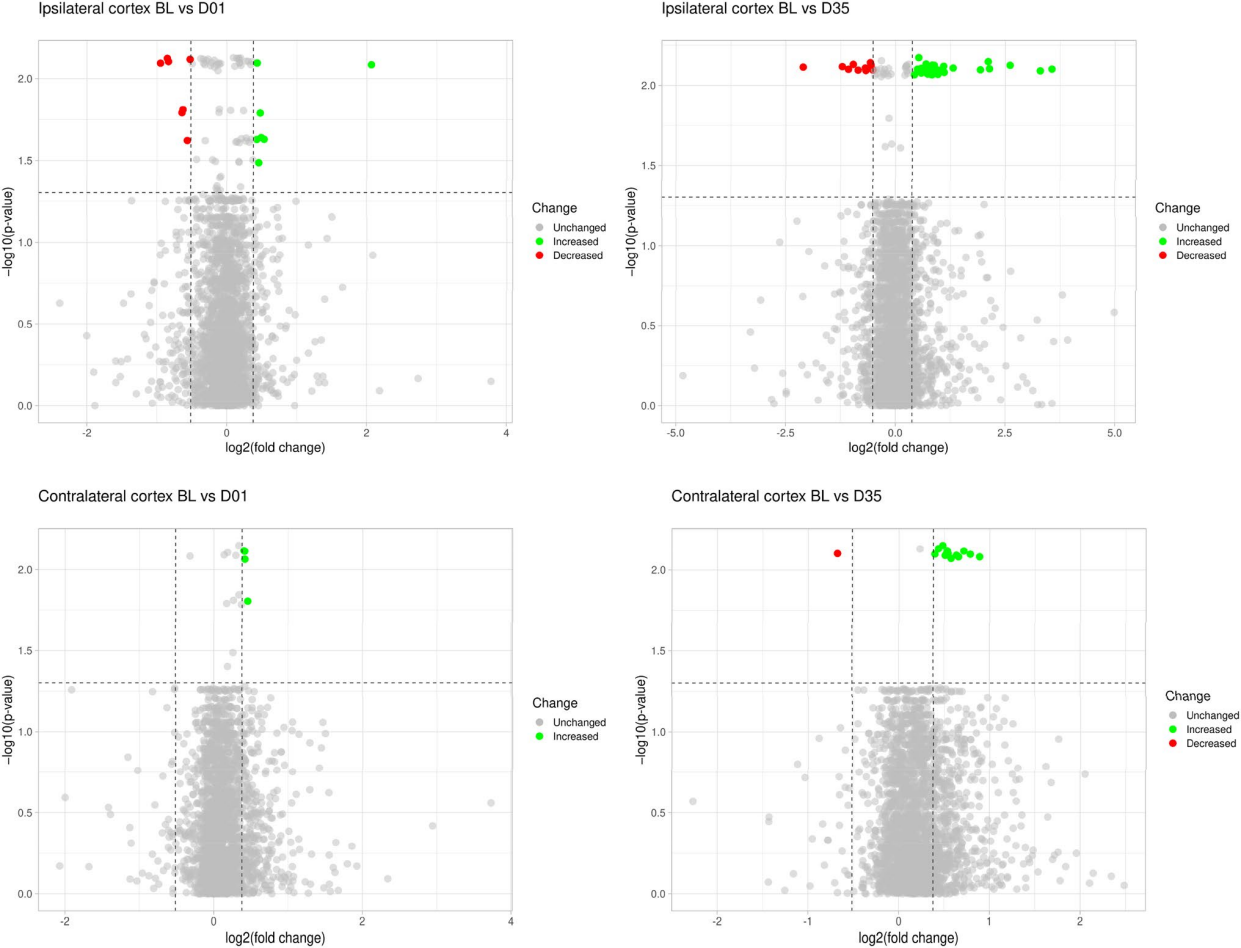
Volcano plots illustrating differentially expressed proteins in the ipsilateral and contralateral cortices. The top left plot shows differentially expressed proteins from ipsilateral cortices between baseline (BL) and day 1 (D01; 7 downregulated and 8 upregulated proteins), while the top right plot shows differentially expressed proteins from IL cortices between BL and day 35 (D35; 12 downregulated and 34 upregulated proteins). The bottom left plot presents differentially expressed proteins from contralateral (CL; 4 upregulated proteins) cortices between BL and D01, while the bottom right plot shows CL cortices between BL and D35 (1 downregulated and 13 upregulated proteins). Fold change thresholds were set at FC < 0.7 for decreased proteins (red dots) and FC > 1.3 for increased proteins (green dots); unchanged proteins are shown in grey. Significance threshold was set for proteins with p-values < 0.05. Volcano plots were generated using the web-based platform VolcaNoseR

To elucidate temporal patterns in protein expression, clustering analyses were conducted for both ipsilateral and contralateral cortices. In the ipsilateral cortex, 74 significantly altered proteins were classified into four expression clusters based on fold change dynamics across time points: proteins that were upregulated in both acute and chronic phases (*acute UP_chronic UP*; *N* = 29), those upregulated acutely and downregulated chronically (*acute UP_chronic DOWN;* *N* = 8), proteins initially downregulated and later upregulated (*acute DOWN_chronic UP;* *N* = 15) and those consistently downregulated over time (*acute DOWN_chronic DOWN;* *N* = 22) (Fig. 2c). In contrast, the 15 altered proteins identified in the contralateral cortices grouped into only two temporal patterns, with the majority showing sustained upregulation throughout the study period (14 proteins) and one protein displaying persistent downregulation (Fig. 2d). This analysis highlights distinct molecular trajectories in the affected and unaffected hemispheres following stroke, suggesting that the contralateral cortex undergoes a more limited but measurable adaptive response. Volcano plots were generated to illustrate protein expression changes in the ipsilateral and contralateral cortices across time points (Fig. 3). A detailed list of increased, decreased, and unchanged DEPs in both ipsilateral and contralateral cortices, along with their distribution across time points, is provided in Supplementary File 1, Supplementary Table 3, and Supplementary Table 4.

**Fig. 4:**
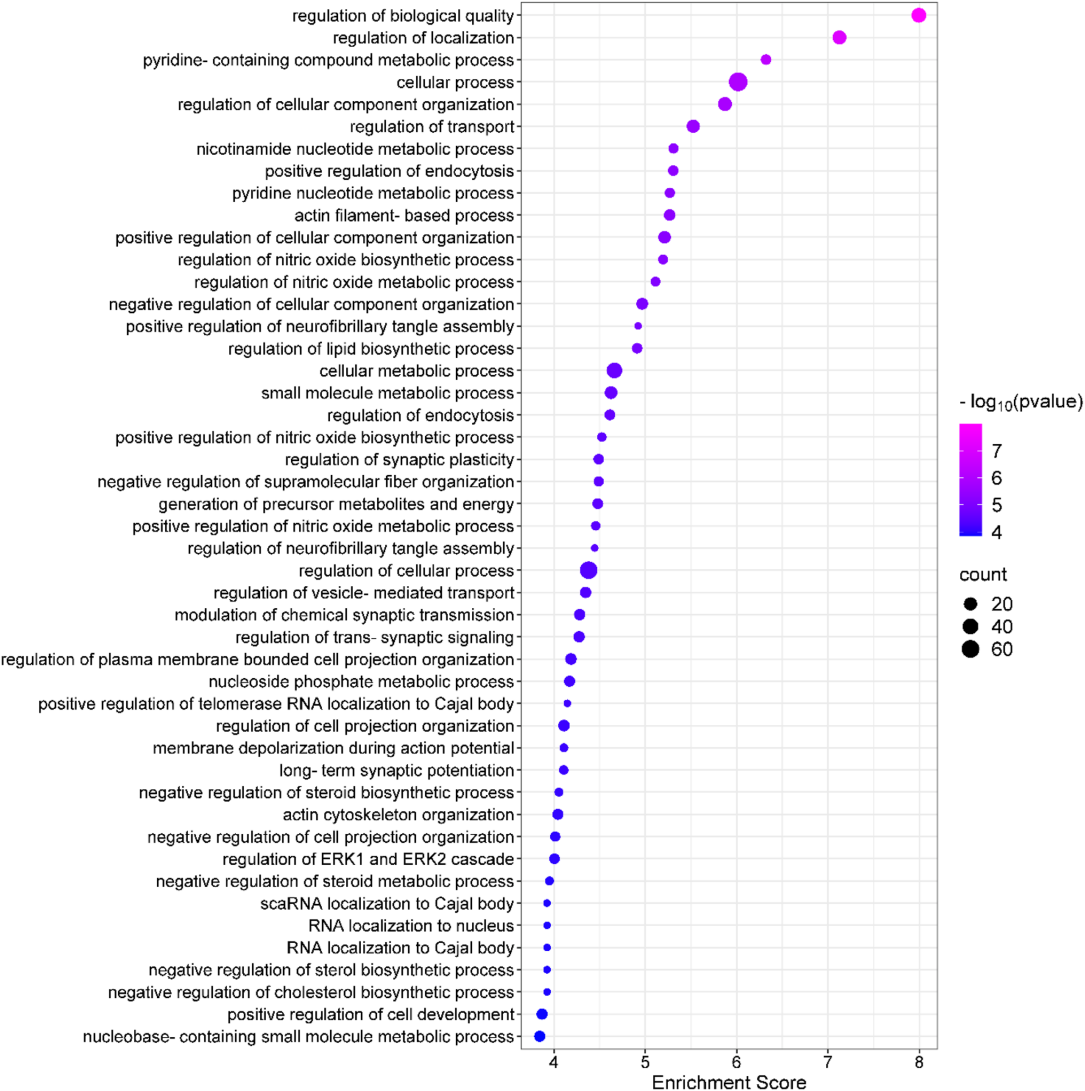
Enrichment analysis of proteomic alterations in the ipsilateral cortex post-stroke. Results of gene ontology analysis conducted for the differentially expressed proteins identified in the ipsilateral cortical tissue, focusing on the “biological process” category. Enrichment scores were calculated as − log10(p-values). The size of each circle represents the number of protein hits associated with each gene ontology term

To further characterize the functional significance of the proteomic changes, gene ontology enrichment analysis was conducted using the PANTHER 19.0 platform. The gene identifiers of the 74 DEPs from the ipsilateral cortex were categorized under the “biological process” classification. All proteins were successfully annotated, except for two instances where multiple mappings were noted (*Tcp1* and *Clu* genes). The identified proteins were distributed across 47 distinct biological processes (Fig. 4). The detailed list of DEPs and their distribution in BP gene ontology terms is provided in Supplementary File 2; Table 6. Among the most enriched biological processes were regulation of biological quality (ES = 7.99), regulation of cellular component organization (ES = 5.87), regulation of transport (ES = 5.53), actin filament-based processes (ES = 5.27), regulation of lipid biosynthetic process (ES = 4.91), and generation of precursor metabolites and energy (ES = 4.48). Additional highly enriched categories included neurofibrillary tangle assembly (ES = 4.45), trans-synaptic signaling (ES = 4.27), cell projection organization (ES = 4.11), and long-term synaptic potentiation (ES = 4.11), among others. Enrichment scores, the number of identified genes, and full gene listings for each biological process are provided in Supplementary File 2, Table 7. Notably, the majority of DEPs were assigned to multiple biological processes, underscoring the complexity and pleiotropic nature of the molecular response to ischemic injury.

To investigate the functional implications of proteomic alterations following ischemic stroke, gene set enrichment analysis (GSEA) was performed using the PANTHER Classification System and the *Mus musculus* reference database. The analysis aimed to identify overrepresented gene ontology (GO) categories (Molecular Function (MF), Cellular Component (CC), and Biological Process (BP)) among DEPs in both ipsilateral and contralateral cerebral cortices. In the ipsilateral cortex, enrichment analysis revealed 47 significantly changed biological processes (Fig. 4), 21 molecular functions, and 56 cellular components associated with the post-ischemic proteomic profile. These findings reflect broad changes in cellular physiology following ischemia and offer insight into the molecular mechanisms involved in tissue injury and recovery. Detailed results of GO MFs and CCs are presented in Supplementary File 1 (Supplementary Figs. 2 and 3). In contrast, proteomic changes in the contralateral cortex did not yield statistically significant enrichment in any of the three GO categories (BP, MF, CC), suggesting a limited or less coordinated molecular response in the unaffected hemisphere.

To further refine the pool of DEPs identified in the ipsilateral cortex, a fold change (FC) threshold was applied to the initial set of 74 proteins. Proteins with FC > 1.3 were classified as upregulated, while those with FC < 0.7 were considered downregulated. This filtering resulted in a subset of 46 DEPs meeting the specific criteria. Of these, 28 proteins were assigned to the *acute UP_chronic UP* cluster, characterized by elevated expression levels in the acute phase, the chronic phase, or both. The identified proteins in this group include argininosuccinate synthase (*Ass1*), atlastin-3 (*Atl3*), carbonic anhydrase 2 (*Ca2*), CD82 antigen (*Cd82*), copine-3 (*Cpne3*), cathepsin D (*Ctsd*), acyl-CoA-binding protein (*Dbi*), m7GpppX diphosphatase (*Dcps*), elongation factor 1-alpha 1 (*Eef1a1*), ectonucleoside triphosphate diphosphohydrolase 2 (*Entpd2*), glutathione S-transferase P 1 (*Gstp1*), histone H1.0 (*H1-0*), histone H4 (*H4c16*), hydroxyacyl-coenzyme A dehydrogenase, mitochondrial (*Hadh*), myelin-oligodendrocyte glycoprotein (*Mog*), unconventional myosin-VI (*Myo6*), profilin-1 (*Pfn1*), 6-phosphogluconolactonase (*Pgls*), purine nucleoside phosphorylase (*Pnp*), dihydropteridine reductase (*Qdpr*), complement C3 (*C3*), clusterin (*Clu*), dynamin-2 (*Dnm2*), glial fibrillary acidic protein (*Gfap*), glycogenin-1 (*Gyg1*), isochorismatase domain-containing protein 1 (*Isoc1*), splicing factor 3 A subunit 3 (*Sf3a3*), and transaldolase (*Taldo1*). FC values for these proteins, which may be involved in early damage mechanisms as well as structural and functional recovery and repair processes, are presented in Supplementary File 1; Supplementary Fig. 4a1–a6.

In the *acute DOWN_chronic DOWN* cluster, 12 proteins met the FC-based downregulation criteria. These included ADP-ribosylation factor GTPase-activating protein 1 (*Arfgap1*), eukaryotic translation initiation factor 4B (*Eif4b*), guanine nucleotide-binding protein subunit beta-5 (*Gnb5*), L-lactate dehydrogenase A chain (*Ldha*), MAP6 domain-containing protein 1 (*Map6d1*), ephexin-1 (*Ngef*), neuroplastin (*Nptn*), protocadherin 1 (*Pcdh1*), protein phosphatase methylesterase 1 (*Ppme1*), sodium channel protein type 2 subunit alpha (*Scn2a*), sodium channel subunit beta-2 (*Scn2b*), and PHD finger protein 24 (*Phf24*), as shown in Supplementary File 1; Supplementary Fig. 4b1–b2. No proteins in the datasets met the FC criteria for the *acute UP_chronic DOWN* cluster. However, six proteins (annexin A1 (*Anxa1*), apolipoprotein E (*Apoe*), macrophage-capping protein (*Capg*), cathepsin Z (*Ctsz*), Ras GTPase-activating-like protein IQGAP2 (*Iqgap2*), and synaptogyrin-1 (*Syngr1*)) were grouped under the *acute DOWN_chronic UP* cluster, as presented in Supplementary File 1; Supplementary Fig. 4c.

In the contralateral cortex, 14 proteins were classified into the *acute UP_chronic UP* cluster using the same FC > 1.3 threshold. These included 1-acyl-sn-glycerol-3-phosphate acyltransferase gamma (*Agpat3*), argininosuccinate synthase (*Ass1*), choline transporter-like protein 2 (*Slc44a2*), D-aminoacyl-tRNA deacylase 1 (*Dtd1*), endonuclease domain-containing 1 protein (*Endod1*), eukaryotic translation initiation factor 4B (*Eif4b*), fructose-2,6-bisphosphatase (*Tigar*), glutamate receptor ionotropic, kainate 2 (*Grik2*), palmitoyl-protein thioesterase 1 (*Ppt1*), protein-arginine deiminase type-2 (*Padi2*), pyridoxine kinase (*Pdxk*), stomatin-like protein 2 (*Stoml2*), UBX domain-containing protein 6 (*Ubxn6*), and voltage-dependent calcium channel gamma-8 subunit (*Cacng8*). Only one protein, tubulin beta-2B chain (*Tubb2b*), was assigned to the *acute DOWN_chronic DOWN* cluster. No proteins in the contralateral cortex satisfied the fold change criteria for the *acute UP_chronic DOWN* or *acute DOWN_chronic UP*. Corresponding fold change values for selected proteins are detailed in Supplementary File 1; Supplementary Fig. 5 (a1–a3 for *acute UP_chronic UP*; a4 for *acute DOWN_chronic DOWN*). Applying defined FC thresholds, in conjunction with robust statistical analyses such as permutation testing and FDR correction, allowed for a more focused interpretation of the proteomic data. This approach helped distinguish proteins exhibiting biologically meaningful expression changes across the acute and chronic phases of stroke. Moreover, among the 74 DEFs in the ipsilateral cortex and 15 DEPs in the contralateral cortex, only two proteins were shared: argininosuccinate synthase (*Ass1*) and eukaryotic translation initiation factor 4B (*Eif4b*).

#### Association of Protein Expression with Ischemic Lesion Size and Functional Recovery

For each identified protein, corresponding data were available at the individual animal level, including measurements of the ischemic lesion size assessed by MRI and neurological deficit determined through standardized scoring. To evaluate potential associations between protein expression and both structural brain damage and functional outcomes, protein FC were plotted as the dependent variable, with ischemic lesion volume and neurological scores serving as independent variables. Differentially expressed proteins were grouped into time-dependent expression clusters. Within each cluster, we highlighted proteins showing a visually apparent linear relationship between their fold-change values and stroke-related parameters, specifically lesion size, changes in lesion progression, and differences in neurological score. This approach facilitated the identification of potential correlations between molecular alterations and stroke-related phenotypes. An overview of the selection criteria and number of DEFs based on FC thresholds, lesion size, acute neurological impairment, and indices of structural reorganization and functional recovery in both hemispheres is shown in Table 1.

**Table 1:**
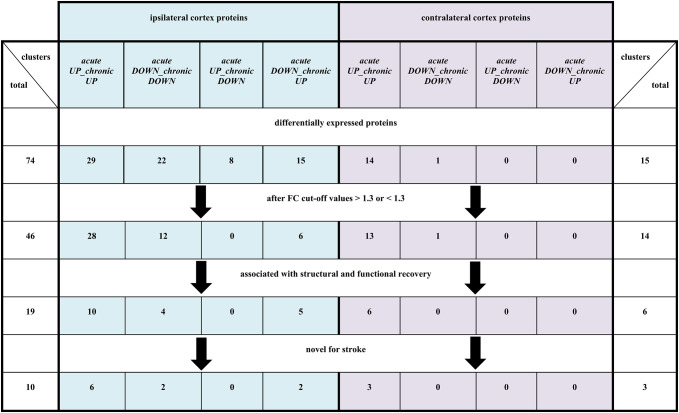
Selection of differentially expressed cortical proteins based on fold change thresholds and their correlation with ischemic lesion size, acute neurological deficit, and indices of functional recovery and structural reorganization in the ipsilateral and contralateral post-ischemic cortices

**Fig. 5:**
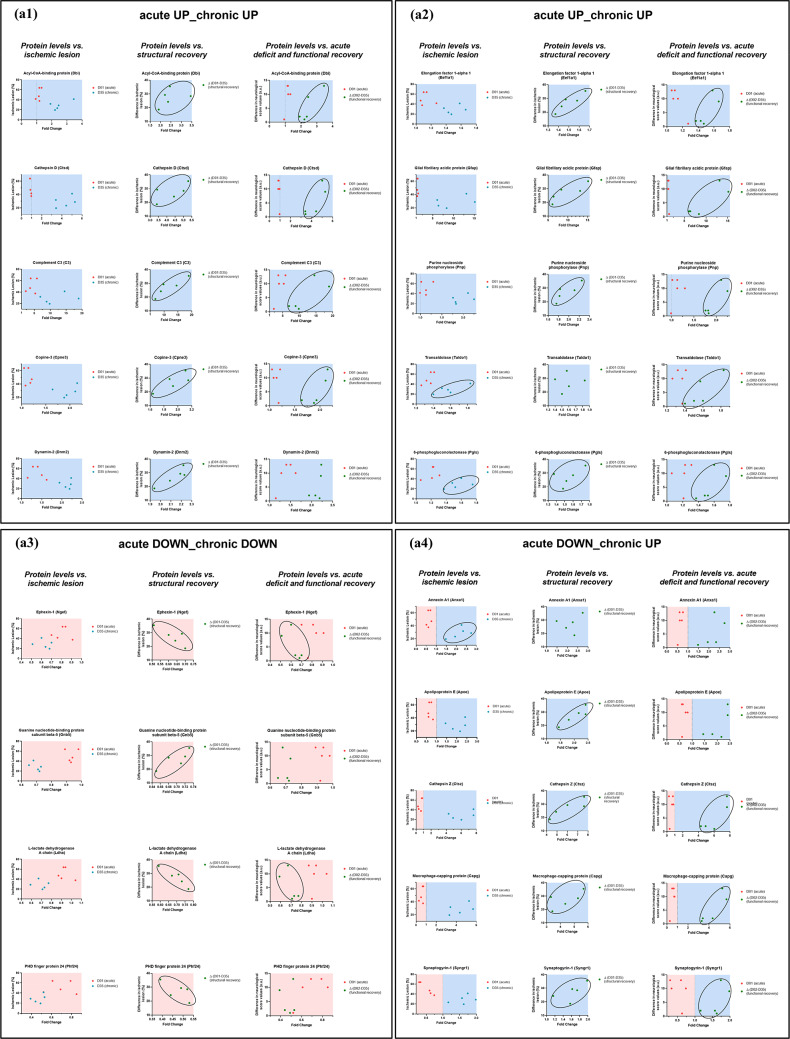

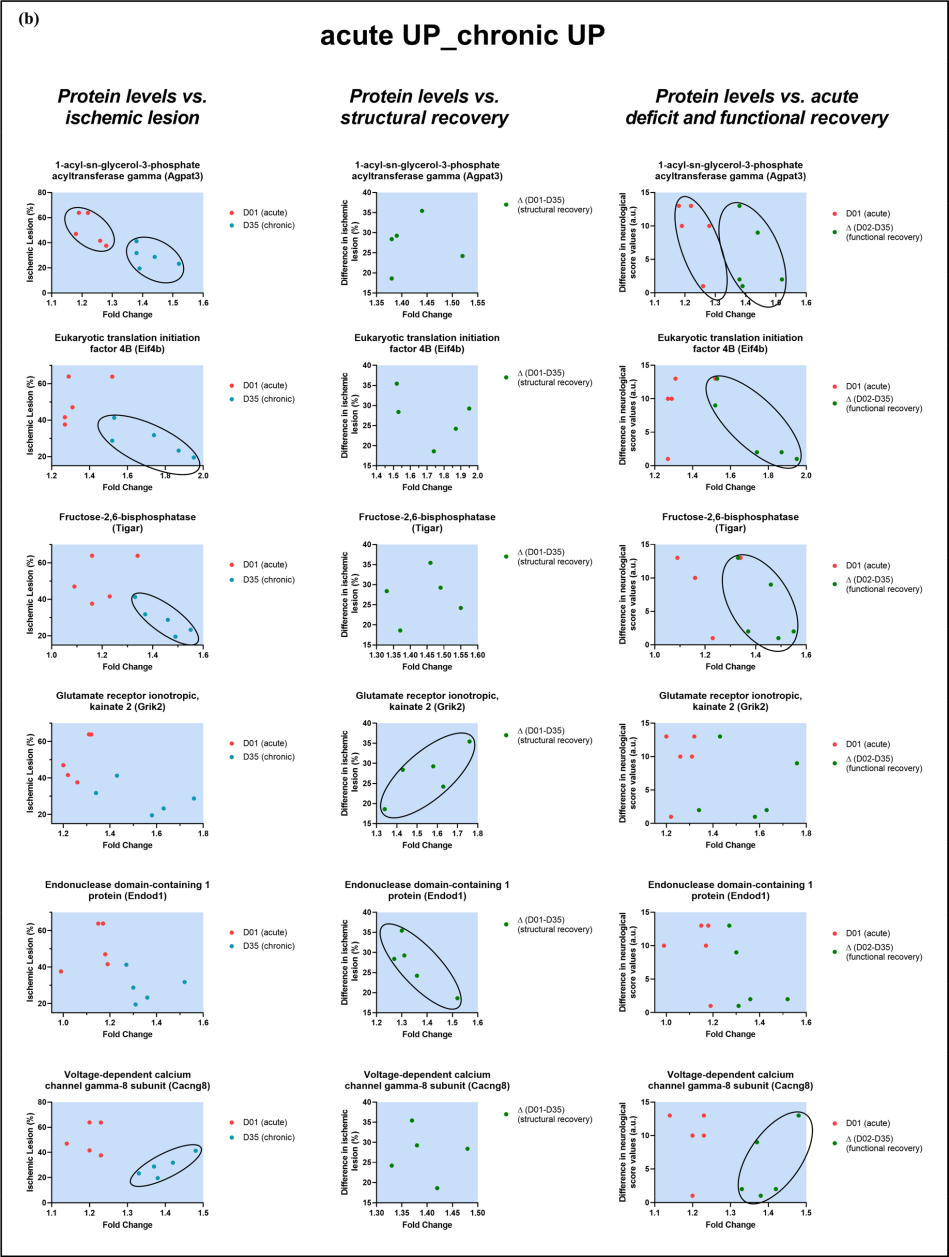
Expression profiles of selected proteins in the ipsilateral (**a1**–**a4**) and contralateral (**b**) cerebral cortex associated with acute ischemic damage and chronic functional and structural recovery. Plots represent fold changes of selected proteins that were significantly upregulated or downregulated in the acute and/or chronic phases following stroke. Selected proteins were categorized into three clusters: acute upregulation with sustained upregulation in the chronic phase (*acute UP_chronic UP*); sustained downregulation (*acute DOWN_chronic DOWN*); and acute downregulation with chronic upregulation (*acute DOWN_chronic UP*). Functional recovery was assessed by changes in neurological scores between the acute and chronic phases, expressed as Δ (D02 − D35). Acute neurological deficits were evaluated as the change from baseline (BL) to D01, denoted as (D01 − BL). Structural reorganization was analyzed by comparing ischemic lesion size between acute and chronic phases, calculated as Δ (D01 − D35), defined by subtracting the percentage of tissue loss observed on day D35 (chronic phase) from the percentage of ischemic lesion in the ipsilateral hemisphere at D01 (acute phase)

In addition to analyzing protein expression at predefined time points (D01 and D35), we further examined associations between expression levels and recovery dynamics, both in terms of structural reorganization and functional recovery. These recovery indices were then compared to FC in protein expression during the chronic phase (relative to baseline levels in control non-ischemic animals; BL group). Despite the limited sample size and the consequent infeasibility of performing robust statistical correlation analyses, the data were nonetheless plotted to explore potential relationships between protein expression levels and recovery parameters (Fig. 5). This approach was taken because several patterns (e.g., complement C3 (*C3*), dynamin-2 (*Dnm2*), purine nucleoside phosphorylase (*Pnp*), L-lactate dehydrogenase A chain (*Ldha*), cathepsin Z (*Ctsz*)) appeared consistently across animals and suggested biologically meaningful associations.

Among the 28 upregulated proteins identified in the ipsilateral *acute UP_chronic UP* cluster, 10 proteins exhibited apparent associations with either MRI-based structural reorganization or improved neurological function. These included acyl-CoA-binding protein (*Dbi*), cathepsin D (*Ctsd*), complement C3 (*C3*), copine-3 (*Cpne3*), dynamin-2 (*Dnm2*), elongation factor 1-alpha 1 (*Eef1a1*), glial fibrillary acidic protein (*Gfap*), purine nucleoside phosphorylase (*Pnp*), transaldolase (*Taldo1*), and 6-phosphogluconolactonase (*Pgls*) (Fig. 5a1−a2). In the *acute DOWN_chronic DOWN* cluster, 4 (ephexin-1 (*Ngef*), guanine nucleotide-binding protein subunit beta-5 (*Gnb5*), L-lactate dehydrogenase A chain (*Ldha*), and PHD finger protein 24 (*Phf24*)) showed correspondence with recovery measures (Fig. 5a3). In the last cluster (*acute DOWN_chronic UP*), 5 out of 6 proteins were associated with ischemic lesion size or recovery measures. These were annexin A1 (*Anxa1*), apolipoprotein E (*Apoe*), cathepsin Z (*Ctsz*), macrophage-capping protein (*Capg*), and synaptogyrin-1 (*Syngr1*), as shown in Fig. 5a4. Furthermore, we observed remarkably similar FC trajectories and graphical profiles for two functionally unrelated proteins, hydroxyacyl-coenzyme A dehydrogenase (*Hadh*) and unconventional myosine-VI (*Myo6*), both from the *acute UP_chronic UP* cluster (see Supplementary File 1, Supplementary Fig. 4a3 and a4).

**Fig. 6:**
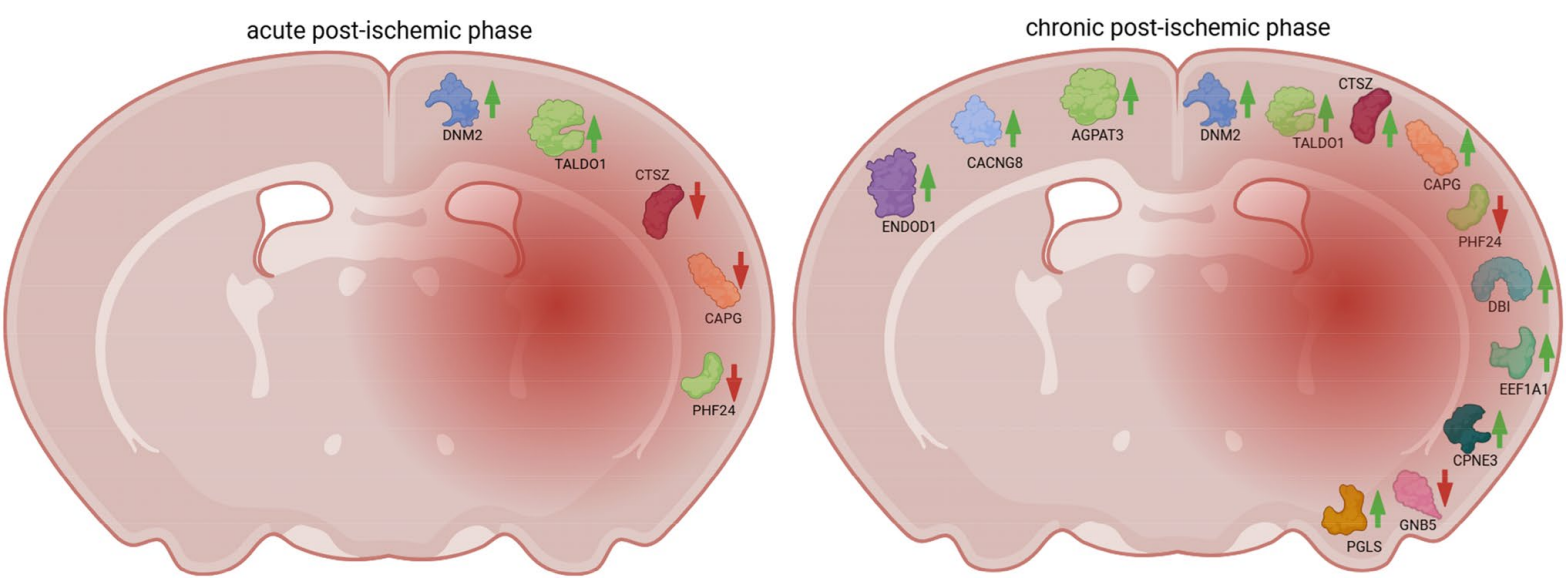
Schematic representation of differentially expressed cortical proteins in acute and chronic post-stroke cortices. 10 proteins, novel to stroke pathophysiology, are identified in the ipsilateral ischemic cortex, and 3 novel proteins in the contralateral cortex. Green upward arrows denote upregulated, while red downward arrows indicate downregulated proteins in each of the post-stroke phases. Created in Biorender.com

In the contralateral cortex, 6 out of 13 proteins in the *acute UP_chronic UP* cluster were associated with recovery measures and lesion size. These proteins included 1-acyl-sn-glycerol-3-phosphate acyltransferase gamma (*Agpat3*), eukaryotic translation initiation factor 4B (*Eif4b*), fructose-2,6-bisphosphatase TIGAR (*Tigar*), glutamate receptor ionotropic kainate 2 (*Grik2*), endonuclease domain-containing 1 protein (*Endod1*), and voltage-dependent calcium channel gamma-8 subunit (*Cacng8*), shown in Fig. 5b. Overall schematic representation of changes in ipsilateral and contralateral cortical proteins novel with stroke pathophysiology can be seen in Fig. 6.

## Discussion

### Cytoskeletal Disruption and Inflammation in Stroke

Ischemic injury in murine cortical tissue induces profound alterations in the organization and function of cytoskeletal structures, including actin filaments, microtubules, and intermediate filaments. These cytoskeletal components are critical not only for maintaining cellular integrity and morphology, but they also play critical roles in intracellular signaling, vesicular transport, and cellular motility. In our study, approximately 13% of the DEPs in the ischemic cortex were associated with cytoskeleton-related processes, emphasizing the central role of cytoskeletal remodeling in neuronal and glial responses to ischemic injury. Among the proteins identified, 8 exhibited significant expression changes and were directly associated with cytoskeletal dynamics. These groups involved core structural proteins such as actin, tubulin, and intermediate filament-associated proteins, as well as regulatory and interacting partners such as glial fibrillary acidic protein (*Gfap*), IQ motif-containing GTPase-activating protein 2 (*Iqgap2*), microtubule-associated protein 6 domain-containing protein 1 (*Map6d1*), and profilin-1 (*Pfn1*). Functional enrichment analysis linked these proteins to biological processes, including endocytosis regulation, modulation of supramolecular fiber organization, actin filament-based processes, actin cytoskeleton organization, vesicle-mediated transport, cellular component organization, and cell projections regulation. Notably, these processes encompass a wide array of neuronal and glial cell functions, both applicable in the acute and chronic phases following ischemic injury. GFAP, a well-studied intermediate filament protein predominantly expressed in astrocytes, showed robust upregulation in both acute (FC = 1.34) and chronic (FC = 9.80) post-stroke phases. Higher GFAP expression during the chronic phase aligns with previous studies reporting a significant increase in GFAP expression within 12 h post-reperfusion in ischemic stroke models (Block et al. 2005), persisting through subacute and chronic stages of ischemia, adding to long-term neuronal loss and reactive gliosis (Feng et al. 2020). Although GFAP is not routinely employed as a clinical biomarker for ischemic stroke, its diagnostic and prognostic potential has been recognized in a range of neurological disorders (Agostini et al. 2021). In experimental MCAO models, GFAP upregulation has been shown to coincide temporally with neuroinflammatory markers, initiating as early as one hour post-injury and persisting for at least one week (Buscemi et al. 2019). These observations support the role of GFAP as a structural component of reactive astrogliosis and a key mediator of the neuroinflammatory process. Furthermore, evidence from prior research suggests that GFAP may exert neuroprotective effects, potentially contributing to brain tissue structural remodeling and recovery (Nawashiro et al. 2000).

Neuroinflammation is a crucial component of ischemic stroke pathology, contributing significantly to secondary brain injury and influencing both acute damage and long-term recovery. The MCAO model in mice effectively replicates the complex inflammatory cascade observed in human stroke, including the activation of resident immune cells such as microglia and astrocytes (Srakočić et al. 2023; Lambertsen et al. 2012; Buscemi et al. 2019). In this study, we identified 7 proteins (*Anxa1*, *Apoe*, *Capg*, *C3*, *Ctsz*, *Entpd2*, *Mog*) whose expression patterns were consistent with immune activation and neuroinflammatory responses in the ischemic brain following MCAO. These proteins exhibit distinct expression profiles, reflecting the dynamic nature of post-ischemic inflammation and its resolution during the chronic phase. Similarly, apolipoprotein E (*Apoe*), a multifunctional protein involved in lipid metabolism and neuronal repair, was downregulated in the acute phase (FC = 0.68) and markedly upregulated in the chronic phase (FC = 2.04). APOE has previously been shown to modulate neuroinflammatory responses and facilitate neuronal regeneration, particularly in the chronic phase of injury (Mahley and Huang 2012). Its upregulation at D35 suggests a reparative role in tissue remodeling and resolution of inflammation, consistent with its established function in neuroprotection and lipid redistribution. In parallel, complement C3 (*C3*), a central component of the complement system, demonstrated robust upregulation at both timepoints (FC = 4.19; FC = 11.79), indicating persistent activation of the innate immune response. The prolonged upregulation of C3 reinforces its role in both acute injury response and sustained inflammation. Its role in the acute and chronic post-stroke phase has been corroborated in preclinical stroke models, where inhibition or genetic deletion of C3 was associated with reduced infarct volumes and improved neurological outcomes (Mocco et al. 2006; Stokowska et al. 2023). Clinically elevated serum C3 levels in ischemic stroke patients were linked to poorer outcomes at three months after ischemia, suggesting C3 as a potential prognostic biomarker (Yang et al. 2021). Macrophage-capping protein (*Capg*), involved in actin filament capping and regulation of immune cell motility, exhibited a biphasic expression pattern, with early suppression at D01 (FC = 0.56) followed by a substantial upregulation at D35 (FC = 4.40). This expression trajectory suggests that CAPG may initially serve to temper excessive inflammation and subsequently participate in phagocytosis and debris clearance during recovery (Chen et al. 2024). Moreover, the lysosomal proteases cathepsin D (*Ctsd*) and cathepsin Z (*Ctsz*) also demonstrated temporally regulated expression, with initial downregulation at D01(FC = 0.89 and 0.55, respectively), and prominent upregulation at D35 (FC = 4.31 and 6.09, respectively). These enzymes are involved in the degradation of cellular debris and have been shown to facilitate tissue repair and neuroprotection in both ischemic and neurodegenerative contexts (Allan et al. 2017; Zhao et al. 2024). Cathepsin D, in particular, is critical for maintaining neuronal survival under ischemic stress and oxygen-glucose deprivation (Hossain et al. 2021), and its protective role extends to other ischemic conditions, as myocardial infarction (Wu et al. 2017). Annexin A1 (*Anxa1*), a glucocorticoid-regulated protein with well-established anti-inflammatory properties, was also upregulated during the chronic phase. ANXA1 plays a critical role in resolving inflammation by inhibiting neutrophil adhesion and transmigration, promoting neutrophil apoptosis, facilitating macrophage-mediated clearance, reducing leukocyte infiltration, and activating neutrophil apoptosis (Sugimoto et al. 2016). Its neuroprotective effects in stroke have been demonstrated through the regulation of microglial polarization, attenuation of pro-inflammatory cytokine production, and inhibition of neuronal apoptosis (Li et al. 2021b, ; Xia et al. 2018, 2022; Xu et al. 2021). The chronic elevation of ANXA1 expression in our model supports its proposed role in orchestrating the resolution of neuroinflammation and promoting recovery.

### Metabolic Dysregulation and Oxidative Stress in Stroke

Ischemic stroke induces profound alterations in cellular homeostasis, including disruptions in the regulation of transcription, translation, and nucleic acid metabolism (Li et al. 2007). Recent proteomic and transcriptomic studies reveal significant changes in the expression of key factors involved in these processes, including transcription factors, translation regulators, and various RNA-binding proteins (Koh 2010). We identified two proteins involved in DNA and nucleotide metabolism, purine nucleoside phosphorylase (*Pnp*) and downregulated guanine nucleotide-binding protein subunit beta-5 (*Gnb5*), whose dysregulation may reflect adaptive or pathological responses to ischemic injury. PNP, an important enzyme of the purine salvage pathway, catalyzes the reversible phosphorolysis of purine nucleosides (such as inosine and guanosine), enabling the recycling of purine bases for nucleotide synthesis (Camici et al. 2023; Somech et al. 2013). In ischemic liver models, elevated PNP activity has been linked to increased reactive oxygen species production, contributing to oxidative damage and impaired organ function (Rao et al. 1990). However, in the context of cerebral ischemia, several preclinical studies have highlighted a neuroprotective role of PNP, potentially through mechanisms involving metabolic adaptation and support to neuronal viability (Tsui et al. 2022; Chojnowski et al. 2021; Thauerer et al. 2012). The observed upregulation of PNP in our dataset may reflect such a protective response aimed at preserving nucleotide pools and facilitating cellular repair during recovery. Conversely, GNB5, which encodes a G protein β subunit involved in GPCR signaling, was downregulated following stroke. Although GNB5 has not been directly implicated in stroke pathogenesis, mutations in this gene are linked to a spectrum of neurodevelopmental and multisystem disorders, including cardiac arrhythmias, cognitive impairment, motor and speech delays, and visual dysfunction, highlighting its essential role in neuronal, cardiac, and autonomic regulation (Lodder et al. 2016; Shamseldin et al. 2016). Given its broad regulatory role in neuronal signaling and homeostasis, the observed reduction in GNB5 expression post-ischemia may reflect disrupted synaptic signaling or impaired intracellular communication in damaged neural circuits. Further studies are needed to elucidate its specific role in ischemic injury.

Following stroke, neuronal tissue undergoes significant metabolic alterations, with the most prominent changes occurring in glucose metabolism. The reduction in glucose and oxygen supply leads to a severe loss of ATP production, disrupting mitochondrial oxidative metabolism and enhancing mitochondria-mediated oxidative stress (Sifat et al. 2022). We identified 4 proteins linked to glucose metabolism that were significantly altered post-stroke: L-lactate dehydrogenase A chain (*Ldha*), 6-phosphogluconolactonase (*Pgls*), glycogenin-1 (*Gyg1*), and transaldolase (*Taldo1*). In our study LDHA levels were slightly downregulated, while previous clinical evidence associates elevated lactate dehydrogenase levels with poor prognosis in ischemic stroke patients, likely reflecting anaerobic glycolysis and tissue damage (Jin et al. 2022). In contrast, GYG1, a self-glycosylating enzyme that plays a key role in glycogen biosynthesis by acting as a primer for the addition of glucose residues from UDP-glucose, was significantly upregulated in both the acute (FC = 1.41) and chronic phases (FC = 2.07). This suggests a potential compensatory shift towards glycogen metabolism in response to stroke-induced energy deficits. In addition, two proteins associated with the pentose phosphate pathways, 6-phosphogluconolactonase (*Pgls*) and transaldolase (*Taldo1*), also showed consistent upregulation. These enzymes contribute to the synthesis of NADPH and nucleotides, which are essential for cellular redox balance and biosynthetic reactions during cellular stress. Upregulation of these enzymes may therefore reflect an adaptive response aimed at enhancing antioxidant defense mechanisms, promoting nucleotide synthesis required for repair and regeneration. Despite their known roles in cellular metabolism, the direct involvement of DBI, PGLS, GYG1, and TALDO1 in ischemic stroke remains scarce, warranting further investigation into their potential roles in stroke pathophysiology.

Furthermore, stroke is closely linked to disruptions in lipid and fatty acid metabolism, which play a critical role in its onset and progression (Haley et al. 2020; Loppi et al. 2022). While several studies have investigated the role of fatty acid-binding proteins (FABPs) in stroke (Guo et al. 2022; Holm et al. 2011), we observed upregulation of acyl-CoA binding protein (*Dbi*) in both acute and chronic phases. DBI is involved in intracellular fatty acid transport and regulation of mitochondrial function. Although no direct evidence currently links DBI to ischemic stroke, its increased expression may support membrane repair, lipid signaling, and mitochondrial energy production during recovery, warranting further investigation.

### Membrane Remodeling and Synaptic Dysfunction in Stroke

Understanding the molecular alterations in neurotransmitter signaling and synaptic plasticity following ischemic stroke is essential for identifying novel proteins that may contribute to neuroregeneration and functional recovery. Stroke-induced neurophysiological changes involve remodeling of the neuronal membrane, synaptic architecture, and neurotransmitter signaling, all of which are regulated by diverse proteins and molecular pathways. In this study, we identified several DEPs involved in membrane trafficking, calcium signaling, protein synthesis, and axon guidance that may contribute to post-stroke recovery mechanisms. One such protein is dynamin-2 (*Dnm2*), a GTPase enzyme involved in membrane trafficking, cytoskeletal dynamics, and intracellular vesicle scission. DNM2 has been implicated in neurite outgrowth and growth cone migration, both of which are critical for neural development and regeneration (González-Jamett et al. 2013, 2014). Although a direct link between DNM2 and stroke pathophysiology remains to be established, disruptions in its associated processes could potentially influence post-stroke recovery mechanisms (González-Jamett et al. 2017). Another protein implicated in stroke recovery is copine-3 (*Cpne3*), which was upregulated in the chronic phase and may be associated with late-stage recovery processes. CPNE3 is a calcium-dependent phospholipid-binding protein involved in signal transduction, cytoskeletal rearrangement, and membrane trafficking. Beyond its established roles in oncogenic cell migration through ErbB2 receptor interaction, CPNE3 has been shown to regulate insulin secretion and glucose uptake in pancreatic β-cells, underscoring its role in β-cell homeostasis (El-Huneidi et al. 2021; Heinrich et al. 2010). Notably, CPNE3 is highly expressed in Schwann cells and has been linked to dysregulation in patients with schizophrenia, Alzheimer’s disease, and individuals exhibiting heightened anxiety and reduced working memory (Khvotchev and Soloviev 2024). These findings suggest that CPNE3 contributes to multiple neurological disorders, although the underlying molecular mechanisms remain largely unknown. The eukaryotic translation elongation factor 1-alpha 1 (*Eef1a1*) also emerged as a protein of potential relevance. Although classically known for its role in delivering aminoacyl-tRNAs to the ribosome during protein synthesis, EEF1A1 has pleiotropic functions that include cytoskeletal organization, apoptosis regulation, and oxidative stress responses. While its direct involvement in stroke pathophysiology has not been extensively studied, its established roles in apoptosis and oxidative stress highlight its potential relevance. Notably, EEF1A1 overexpression has been shown to confer resistance to apoptosis induced by growth factor withdrawal and endoplasmic reticulum stress (Talapatra et al. 2002). More recently, elevated EEF1A1 expression was found to enhance collateral sprouting of corticospinal tract neurons following unilateral pyramidotomy injury, suggesting a role in neuroplasticity and post-injury recovery (Romaus-Sanjurjo et al. 2022). In contrast, we observed downregulation of ephexin-1 (*Ngef*) in both acute and chronic stroke phases. NGEF is a guanine nucleotide exchange factor that regulates Rho GTPase signaling, playing a crucial role in axon pathfinding and synaptic homeostasis (Kim et al. 2019). Recent evidence links NGEF to remyelination processes (Liu et al. 2022a, b) and to blood-brain barrier disruption following cerebral ischemia (Chen et al. 2018a). Thus, its downregulation may reflect impaired axonal regeneration and compromised vascular integrity in the ischemic brain. Another protein involved in synaptic function is synaptogyrin-1 (*Syngr1*), a synaptic vesicle membrane protein that modulates synaptic plasticity and participates in the synaptic vesicle exo-endocytic cycle (Janz et al. 1999; Stevens et al. 2012). SYNGR1 was found to be differentially expressed in our dataset, and previous studies have shown its downregulation correlates with improved motor recovery in levodopa-treated Parkinsonian models, suggesting that reduced SYNGR1 expression may be beneficial under certain neurodegenerative or neuroplastic conditions (Häggman Henrikson et al. 2020). Lastly, PHD finger protein 24 (*Phf24*), also known as GEC1-interacting protein (GECIP), was identified as a protein with putative involvement in neurotransmission. PHF24 is expressed in GABAergic inhibitory interneurons, and it’s thought to modulate GABAergic signaling in the central nervous system (Numakura et al. 2021). Although, there are some findings that Phf24-null rats exhibited increased sensitivity to chemically induced seizures, emotional hyper-reactivity, and cognitive deficits indicating that PHF24 is crucial for proper central nervous system function, particularly in preventing epileptogenesis and regulating emotional and cognitive behaviors (Serikawa et al. 2019), no correlation between stroke and PHF24 protein was observed in literature.

### Contralateral Cortical Proteome Response Following Ischemia

The contralateral hemisphere is increasingly recognized as a critical contributor to functional compensation and motor recovery following ischemic stroke, particularly in cases where damage to the ipsilateral motor cortex and corticospinal tract is substantial. Enhanced activity as a form of neuroplastic adaptation in the contralateral cortex has been observed both in preclinical models and human patients and is associated with improved outcomes (Buetefisch 2015; Riecker et al. 2010). Metabolomic and transcriptomic analyses have revealed alterations in various biomacromolecules, including proteins, lipids, and mRNA across both hemispheres, underscoring the bilateral nature of ischemia-induced neurobiological remodeling (Filippenkov et al. 2023; Rakib et al. 2019; Demyanenko et al. 2018). In the present study, we identified several proteins with altered expression in the contralateral cortex that may contribute to post-stroke metabolic and synaptic reorganization. Among these, 1-acyl-sn-glycerol-3-phosphate acyltransferase gamma (*Agpat3*) was upregulated. AGPAT3 plays a crucial role in lipid biosynthesis, catalyzing the acylation of lysophosphatidic acid to phosphatidic acid, an essential intermediate in the synthesis of glycerophospholipids and triacylglycerols, which are vital for maintaining proper cellular lipid composition (Yuki et al. 2009). Even though lipid metabolism is known to be profoundly affected by ischemia, the specific role of AGPAT3 in stroke remains unexplored. Its upregulation may reflect adaptive lipid remodeling processes that support membrane repair and cellular survival. Another enzyme found to be upregulated was TP53-induced glycolysis and apoptosis regulator (*Tigar*), also known as fructose-2,6-bisphosphatase. TIGAR functions as a metabolic regulator by modulating glycolytic flux and reducing ROS production. In the context of cerebral ischemia, TIGAR has been shown to exert neuroprotective effects by reducing oxidative stress, supporting mitochondrial function, and mediating downregulation of pro-inflammatory markers such as iNOS, COX-2, IL-1β, and TNF-α in astrocytes (Li et al. 2014, 2021a; Chen et al. 2018a; Zhou et al. 2016; Liu et al. 2022b). The upregulation of TIGAR may represent an adaptation aimed at mitigating secondary injury and promoting cellular resilience during enhanced activity. Alternations in translation control mechanisms were also evident, as reflected by the differential expression of eukaryotic translation initiation factor 4B (*Eif4b*). EIF4B is involved in the early steps of mRNA translation initiation, stimulating the unwinding of mRNA to allow ribosomal scanning and initiation (Raught et al. 2004). Decreased EIF4B expression has been previously correlated with stress granule formation, cytoplasmic ribonucleoprotein aggregates that accumulate in response to cellular stress, including ischemia (Ayuso et al. 2016). Additionally, two upregulated proteins related to pre- and postsynaptic actions of neurotransmitters were identified: glutamate receptor ionotropic kainate 2 (*Grik2*) and voltage-dependent calcium channel gamma-8 subunit (*Cacng8*). GRIK2 encodes a subunit of kainate-type glutamate receptors and has been implicated in excitotoxicity, a process in which excessive glutamate release during ischemia causes harmful calcium influx, leading to neuronal injury and death (Zhu et al. 2014). In contrast, no stroke-related literature was found for CACNG8, a protein primarily recognized as an auxiliary subunit of AMPA-type glutamate receptors (Bai et al. 2022). The final protein identified with altered expression in the contralateral cortex was endonuclease domain-containing 1 protein (*Endod1*), which functions as a tumor suppressor, particularly in prostate cancer, and is implicated in innate immune responses (Qiu et al. 2017). Although ENDOD1 has not been directly associated with stroke or ischemia, the established involvement of other endonuclease-related proteins (e.g., endonuclease VIII-like 1 and endonuclease G) in ischemic injury suggests a potential, yet unexplored, relevance in cerebral ischemia (Zhang et al. 2007; Canugovi et al. 2012). Modest proteomic alterations observed in the contralateral cortex, including the upregulation of Agpat3, Cacng8, and Endod1, likely represent compensatory transhemispheric neuroplasticity, however, contributions from systemic stress responses or procedural influences cannot be excluded.

### Limitations of the Study

While our findings provide important insights into proteomic dynamics following stroke, several limitations warrant discussion. A key limitation is the absence of a sham-operated group, which could further isolate the effects of anesthesia and vessel manipulation. Evidence from other ischemic or MCAO models suggests that sham surgery in the acute phase of stroke is not entirely proteomically inert. For example, Candamo-Lourido et al. (2024) detected 98 dysregulated proteins in sham versus naïve rats after ECA MCAO and 57 overlapping with ischemic groups, indicating that surgery itself can induce measurable changes within 24 h (Candamo-Lourido et al. 2024). Similarly, Simats et al. (2018) observed 47 altered cerebrospinal fluid after sham surgery, with 22 overlapping ischemic signatures (Simats et al. 2018). In contrast, Shao et al. (2017) reported that sham-operated animals had a proteome quite similar to naïve animals one hour after hypoxia-ischemia, indicating modest proteomic perturbations (Shao et al. 2017). Moreover, Gu et al. (2021) found that 71–80% of stroke-altered protein (stroke vs. sham) were also identified when stroke animals were compared to intact controls, indicating substantial concordance between these control strategies in the acute phase (Gu et al. 2021). These findings highlight that sham procedures contribute their own molecular footprint and that both sham and naïve controls are informative. As there are, to our knowledge, no published studies directly comparing sham to naïve animals in the same MCAO model, future studies should include both control types to better disentangle parsing procedure-related from ischemia-specific proteomic changes. The ipsilateral and contralateral cortices were analyzed on different mass-spectrometry devices, although they were the same model, which precluded a reliable direct hemisphere-to-hemisphere proteomic comparison at each time point. Therefore, our analysis primarily focused on temporal changes within each hemisphere rather than on inter-hemispheric differences. Future studies processing both hemispheres simultaneously on the same analytical platform will be required to determine stroke-specific versus remote compensatory molecular responses. Furthermore, proteomic analysis was conducted on the entire ipsilateral cortex without spatial distinction between infarct-core and peri-infarct regions. While this strategy facilitated a comprehensive assessment of global cortical responses to ischemia, it may have obscured region-specific molecular alterations. Future investigations employing spatially resolved or region-targeted proteomic approaches will be necessary to define molecular signatures unique to the infarct core and peri-infarct zones. As our study was designed as a discovery-driven proteomic screen, the functional relevance of several newly identified proteins remains to be validated. Follow-up studies using targeted in vitro and in vivo experiments will be crucial to elucidate the mechanistic roles of these proteins in neuroinflammation, synaptic remodeling, and metabolic adaptation during post-stroke recovery. Although no stroke studies have specifically examined its impact on the proteins identified here, findings from other experimental contexts indicate that opioids can produce modest, region- and protein-specific effects on glial morphology, myelin protein expression, and neuronal transcription factors such as CREB and BDNF (Ryu et al. 2021; Xhakaza et al. 2021). These observations suggest that the potential influence of analgesia on acute-phase proteomic data is likely minor and protein-dependent, and, to our knowledge, none of the novel stroke-related proteins identified in our study have been previously linked to buprenorphine treatment. Finally, a limitation of our cluster approach is that proteins were highlighted based on visual analysis of patterns of fold-change in relation to stroke-related parameters, rather than on formal statistical correlation tests. This qualitative strategy was chosen because of the limited sample size and should be addressed in future studies with larger cohorts.

In conclusion, we defined 13 previously unexplored proteins in the cerebral cortex of mice subjected to ischemic stroke using the Koizumi MCAO model with chronic hypoperfusion. Protein candidates were selected based on statistically significant expression changes, defined fold-change thresholds, association with structural and functional recovery, and absence of prior description in stroke pathophysiology. This targeted approach allowed us to highlight novel molecular players that may contribute to structural reorganization and functional recovery following cerebral ischemia. Among the 13 proteins identified, 10 were differentially expressed in the ipsilateral cortex, including: acyl-CoA-binding protein (*Dbi*), copine-3 (*Cpne3*), dynamin-2 (*Dnm2*), elongation factor 1-alpha 1 (*Eef1a1*), transaldolase (*Taldo1*), 6-phosphogluconolactonase (*Pgls*), guanine nucleotide-binding protein subunit beta-5 (*Gnb5*), PHD finger protein 24 (*Phf24*), cathepsin Z (*Ctsz*), and macrophage-capping protein (*Capg*). An additional three proteins were upregulated in the contralateral cortex: 1-acyl-sn-glycerol-3-phosphate acyltransferase gamma (*Agpat3*), voltage-dependent calcium channel gamma-8 subunit (*Cacng8*), and endonuclease domain-containing 1 protein (*Endod1*) from the contralateral cortex. These proteins lay the groundwork for further mechanistic studies, particularly given their potential involvement in neuroinflammation, synaptic plasticity, metabolic adaptation, and cytoskeletal remodeling after stroke. Furthermore, a deeper understanding of the molecular mechanisms involving these proteins may also uncover novel targets for therapeutic intervention, ultimately contributing to the development of strategies that promote neural repair and improve functional outcomes after stroke.

## Supplementary Information

Below is the link to the electronic supplementary material.

Supplementary material 1 (PDF 1282.5 kb)

Supplementary material 2 (XLSX 1164.2 kb)

## Data Availability

The mass spectrometry proteomics data have been deposited to the ProteomeXchange Consortium via the PRIDE partner repository with the dataset identifier PXD067155.
