# Supplementary material for: Proteomic Analysis of Mouse Cerebral Cortex Following Experimental Ischemic Stroke: Identifying Novel Biomarkers of Damage and Repair
Source: Cell Mol Neurobiol. 2025 Dec 4;46:8. doi: 10.1007/s10571-025-01645-y (PMC12796063; doi:10.1007/s10571-025-01645-y)
Supplement: Supplementary file 1 — Supplementary material 1 (PDF 1282.5 kb) [file 10571_2025_1645_MOESM1_ESM.pdf]

Supplementary Information (Supplementary File 1) for

Proteomic Analysis of Mouse Cerebral Cortex Following Experimental Ischemic Stroke: Identifying Novel Biomarkers of Damage and Repair

Dominik Hamer[1], Ana Butorac[2,3], Daniela Petrinec[1], Monika Berecki[1], Vera M. Mendes[4], Bruno Manadas[4], Vanja Kelava[2], Branimir K. Hackenberger[5], Anton Glasnović[1], Marija Lovrić[2], Srećko Gajović[1*], Marina Dobrivojević Radmilović[1]

[1] University of Zagreb School of Medicine; Croatian Institute for Brain Research, Department of Histology and Embryology, BIMIS – Biomedical Research Center Šalata, 10000 Zagreb, Croatia
[2] BICRO BIOCentre Ltd., 10000 Zagreb, Croatia
[3] Selvita Ltd., 10000 Zagreb, Croatia
[4] CNC - Center for Neuroscience and Cell Biology - UC Biotech - Parque Tecnológico de Cantanhede, 3060-197 Cantanhede, Portugal
[5] Department of Biology, Josip Juraj Strossmayer University of Osijek, 31000 Osijek, Croatia

*** Correspondence:**
Prof. Srećko Gajović, MD, PhD
Acting Head BIMIS – Biomedical Research Center Šalata
University of Zagreb School of Medicine
10000 Zagreb, Šalata 3, Croatia
srecko.gajovic@mef.hr

1 Supplementary Figures and Table

1.1 Supplementary Figures

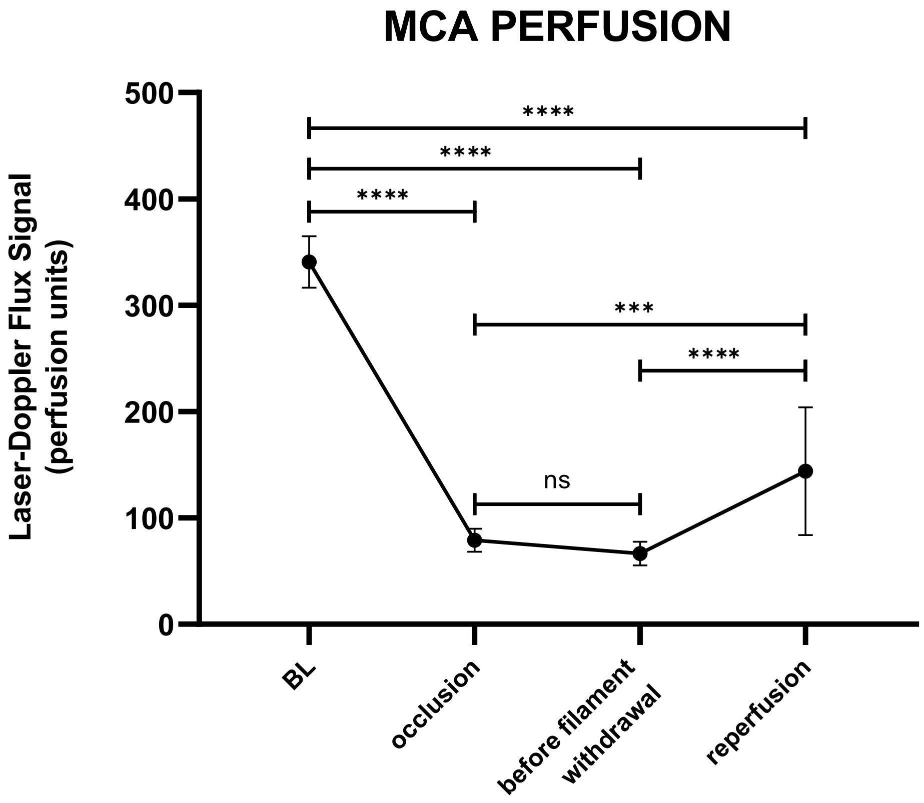

Supplementary Figure 1 Temporal dynamics of blood flow during MCAO induced by the modified Koizumi method. Intraoperative laser Doppler flowmetry reveals incomplete reperfusion following middle cerebral artery occlusion (MCAO) using the Koizumi method of filament insertion via the common carotid artery (CCA). Perfusion was measured before CCA occlusion, immediately after MCA occlusion, before filament withdrawal, and at reperfusion. Statistical analysis was performed using one-way ANOVA followed by Bonferroni *post hoc* test. *** $p < 0.0005$, **** $p < 0.0001$

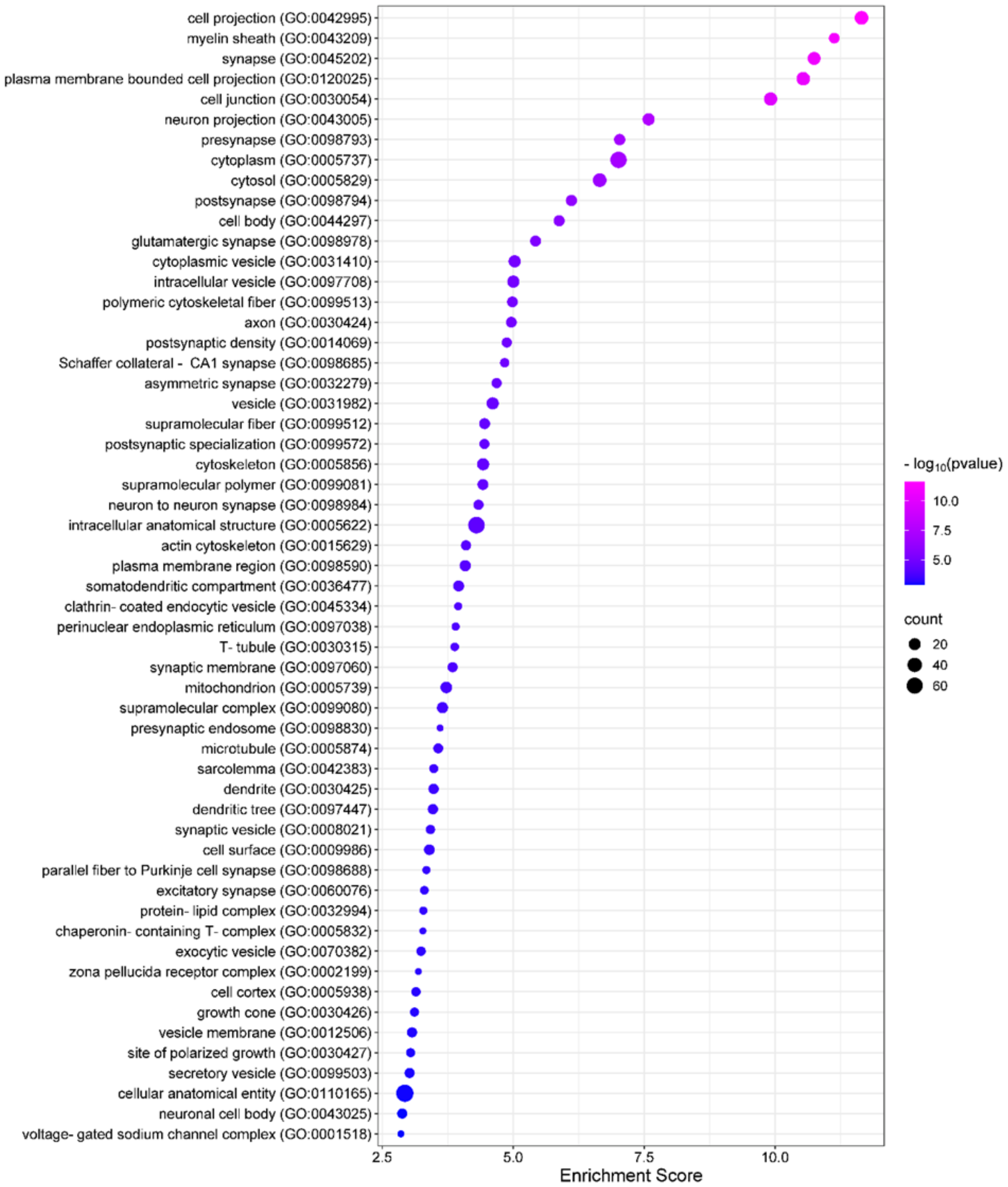

Supplementary Figure 2 Gene ontology enrichment analysis of proteomic alterations in the ipsilateral mouse cortex. Gene ontology (GO) analysis was conducted for the category “ Molecular Function” to characterize proteomic changes in the ipsilateral cortices following middle cerebral artery occlusion. Enrichment scores were calculated as −log10(p-values). The size of each circle corresponds to the number of protein hits associated with the respective GO term

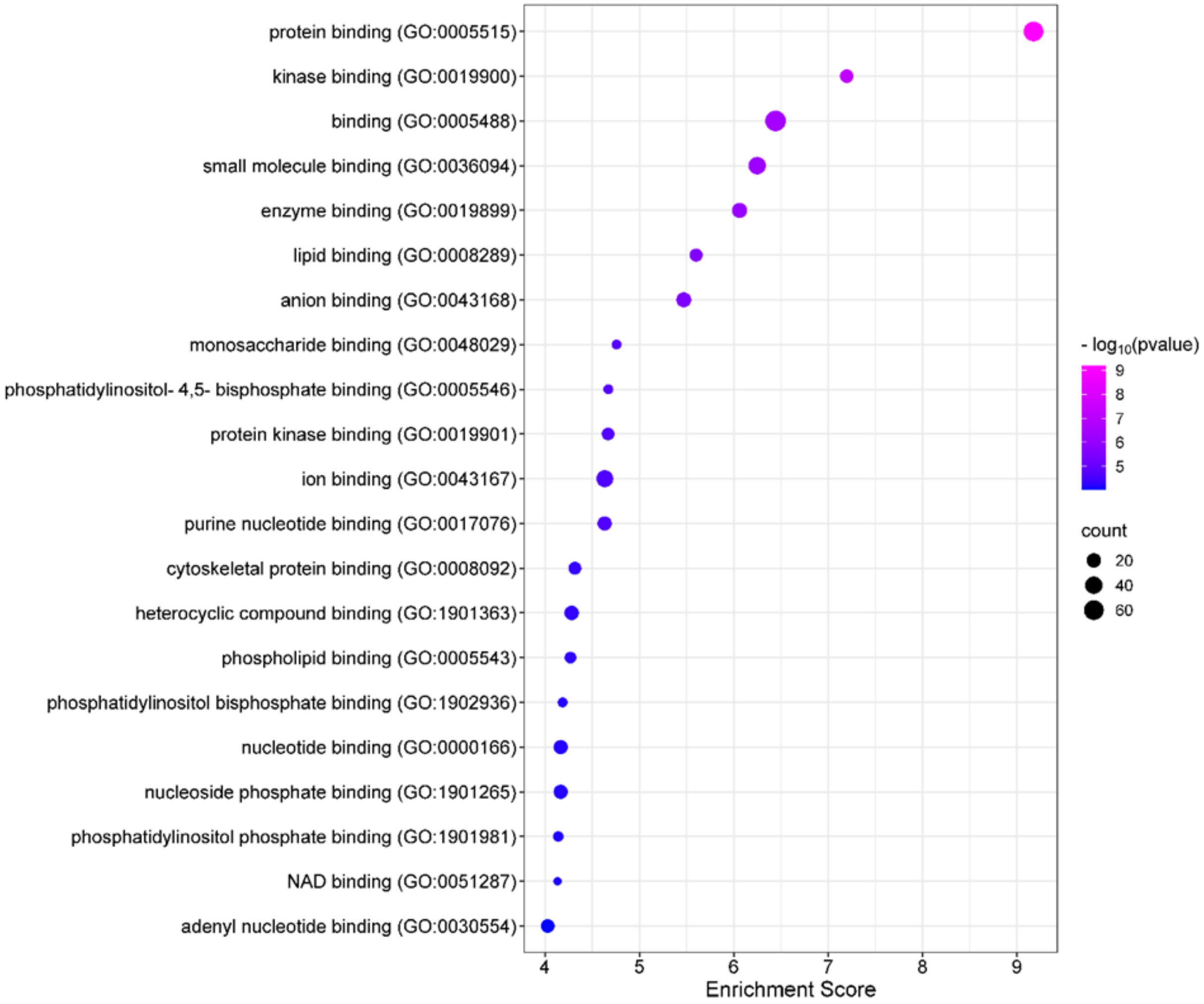

Supplementary Figure 3 Gene ontology enrichment analysis for proteome changes in the ipsilateral mouse cortex. Proteomic alterations in the ipsilateral cortices were analyzed using gene ontology (GO) for the category of “Cellular Component”. Enrichment scores were calculated as $-\log_{10}$(p-values). Circle size represents the number of protein hits in each gene ontology term. The size of each circle corresponds to the number of protein hits associated with the respective GO term

(a1) **acute UP_chronic UP**

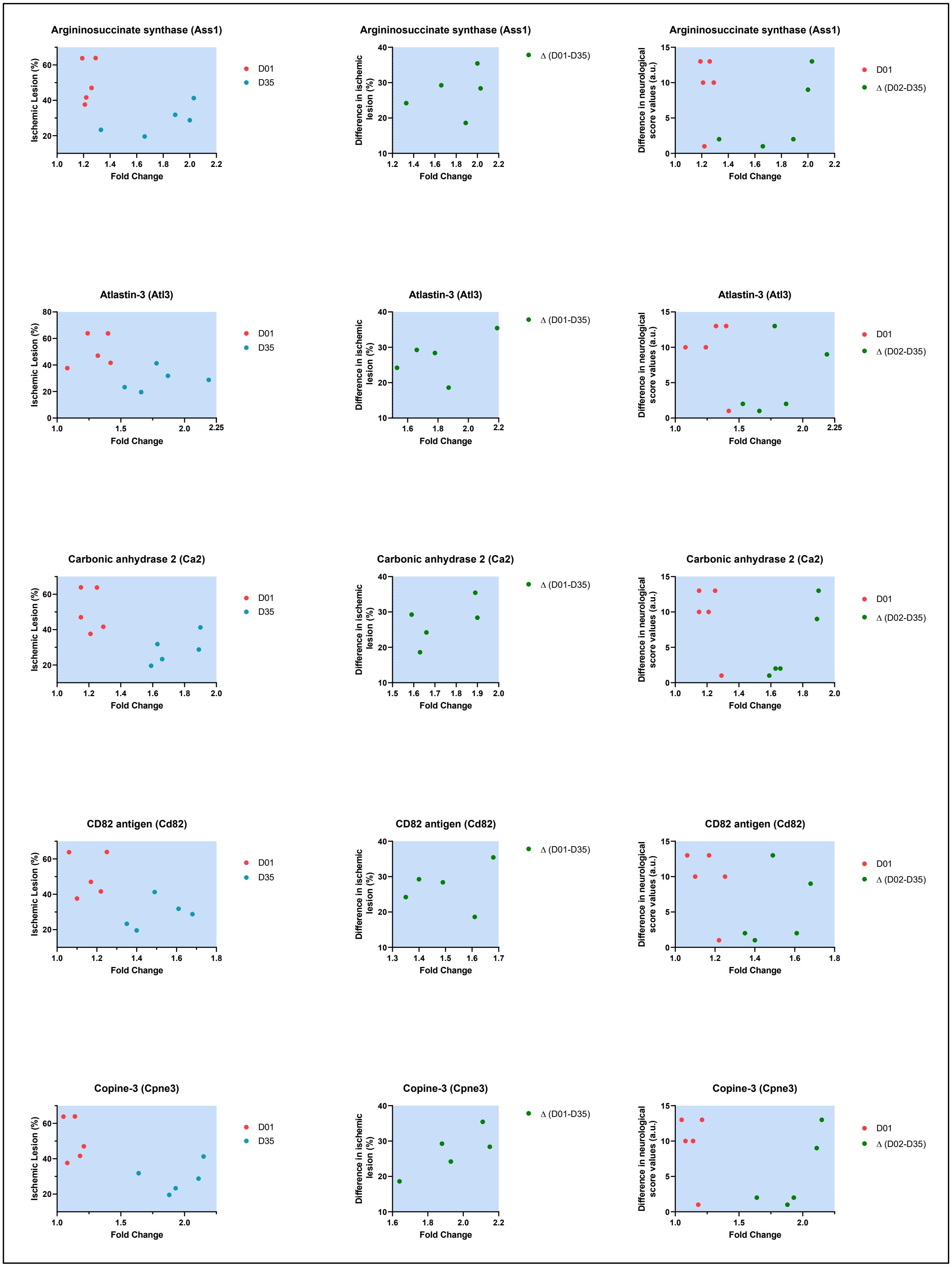

(a2) **acute UP_chronic UP**

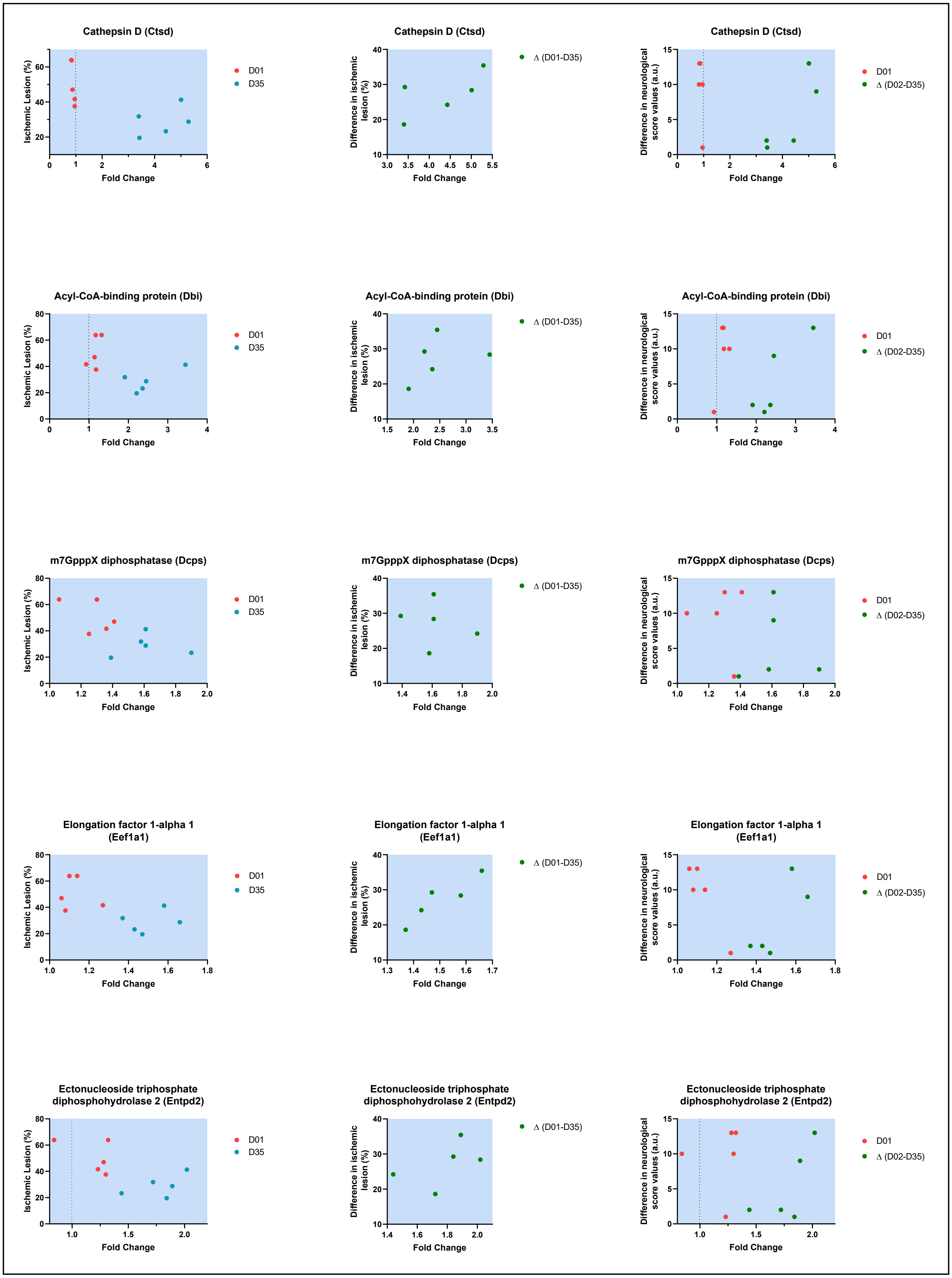

(a3) **acute UP_chronic UP**

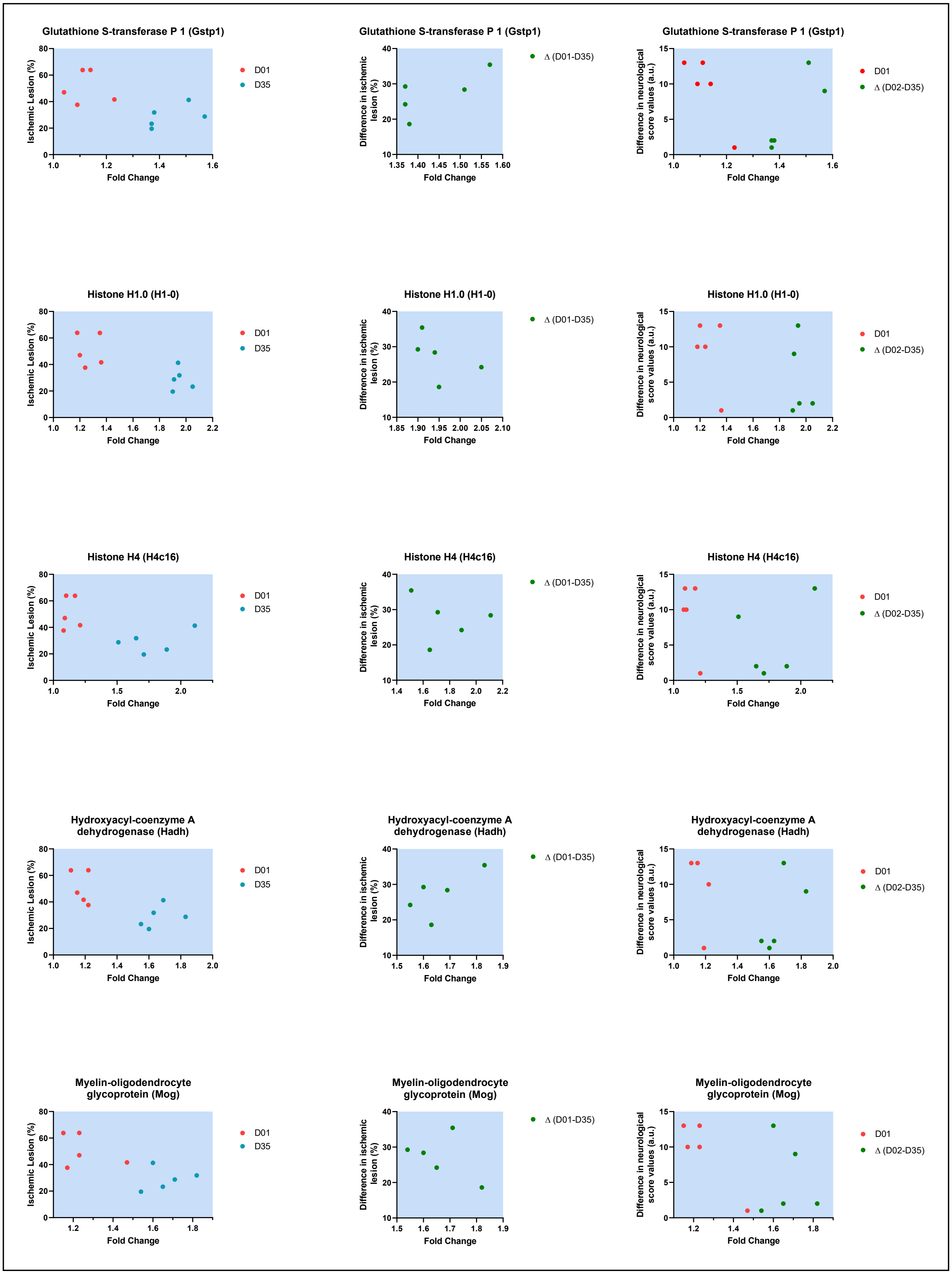

(a4) **acute UP_chronic UP**

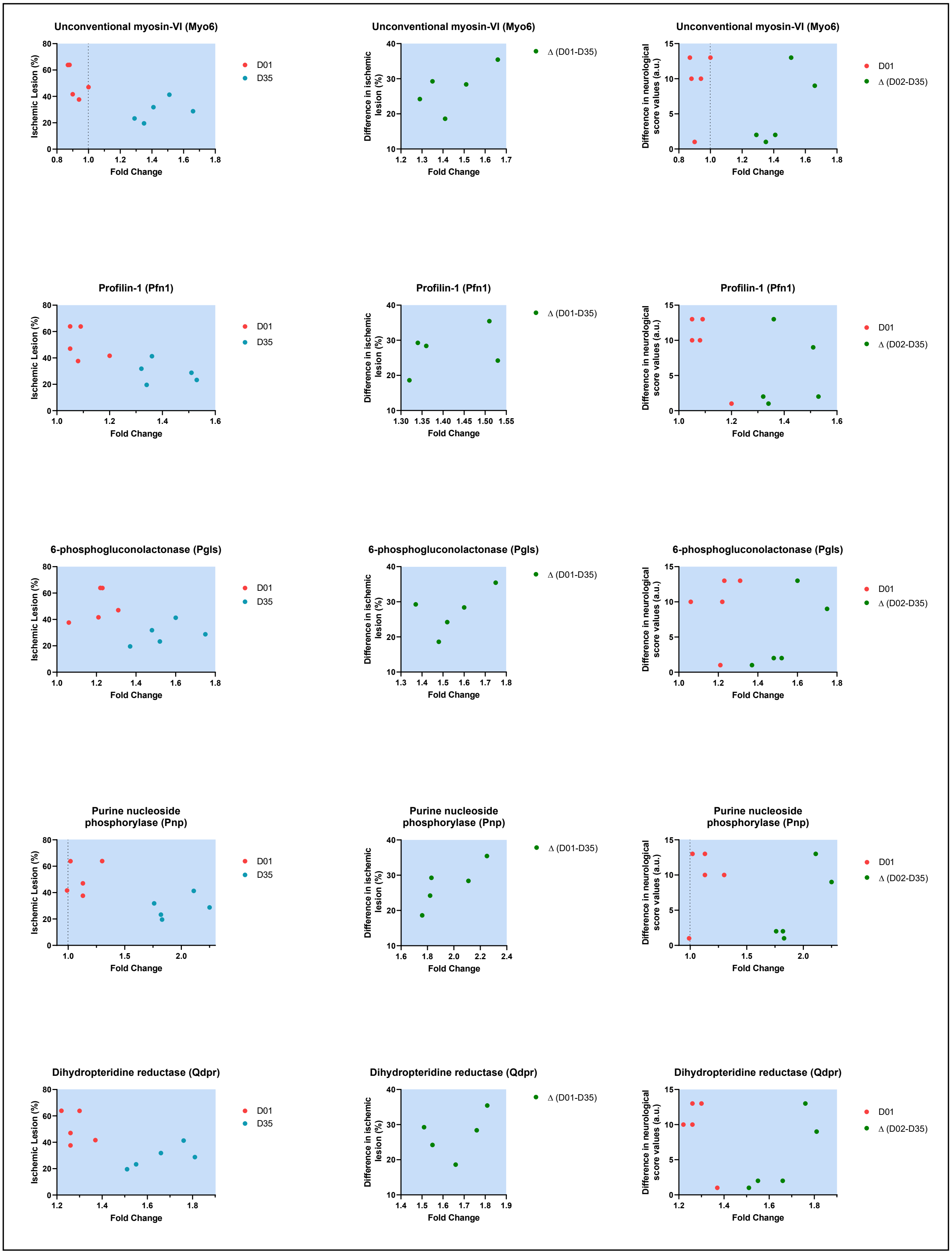

(a5)

acute UP_chronic UP

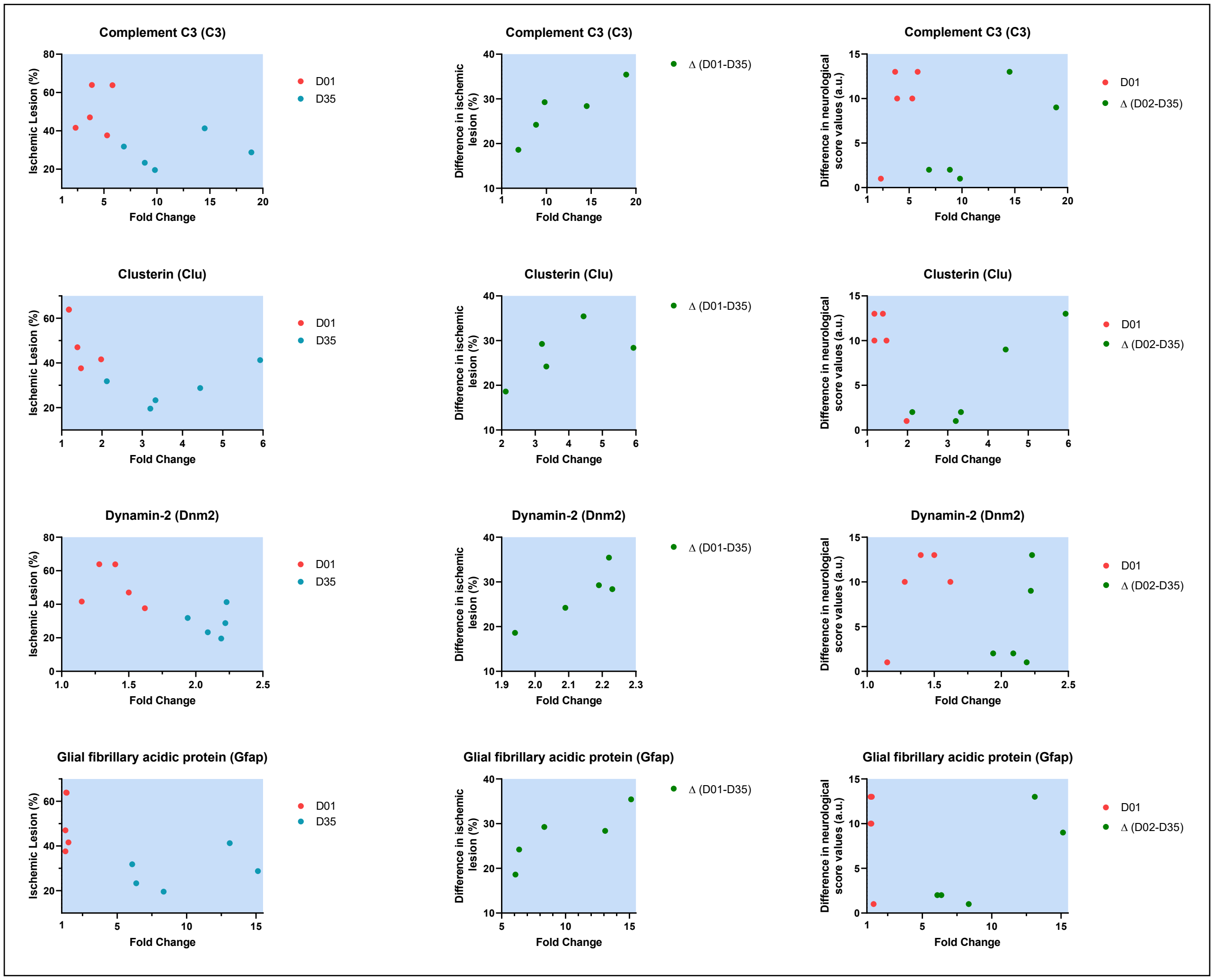

(a6)

acute UP_chronic UP

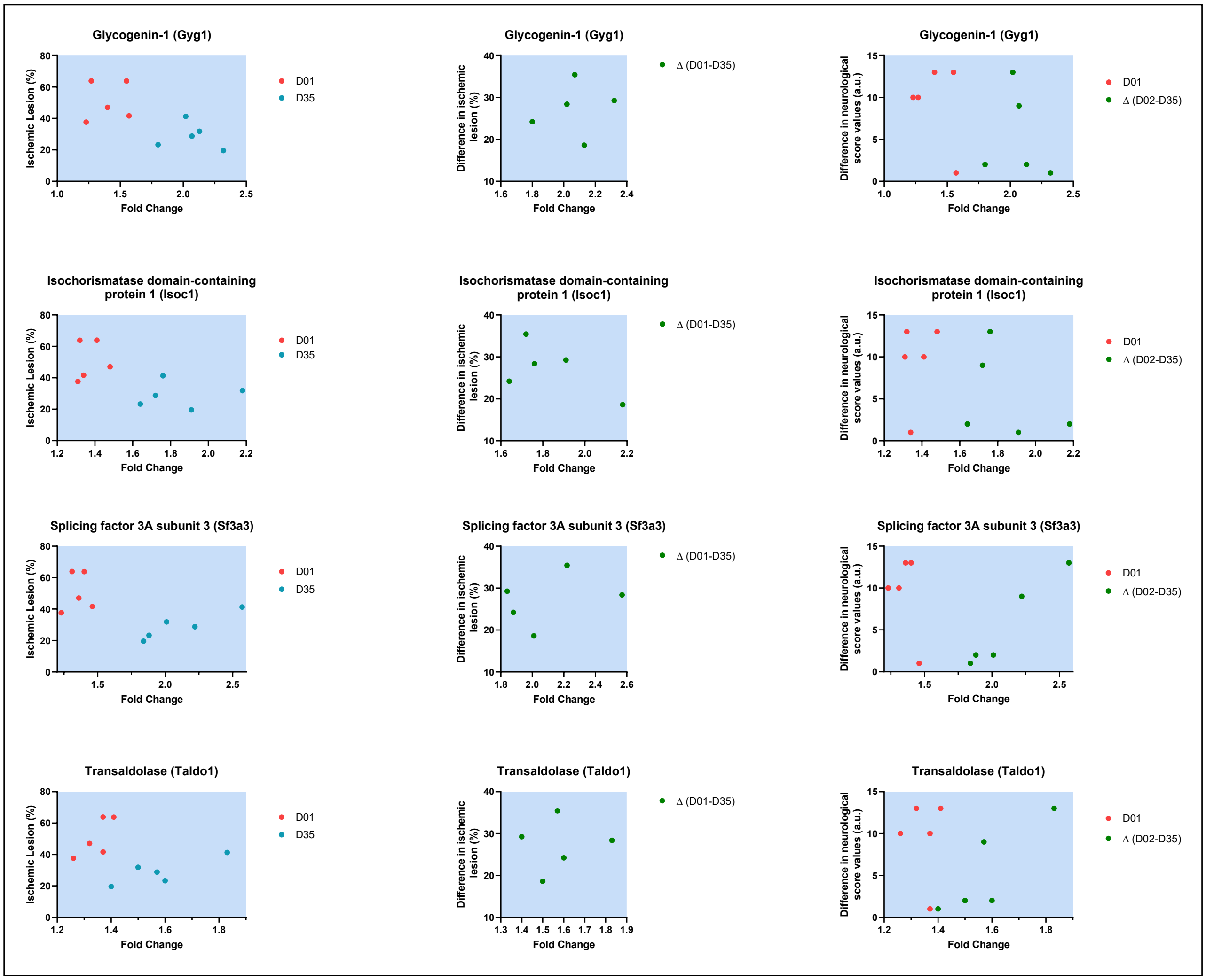

(b1) **acute DOWN_chronic DOWN**

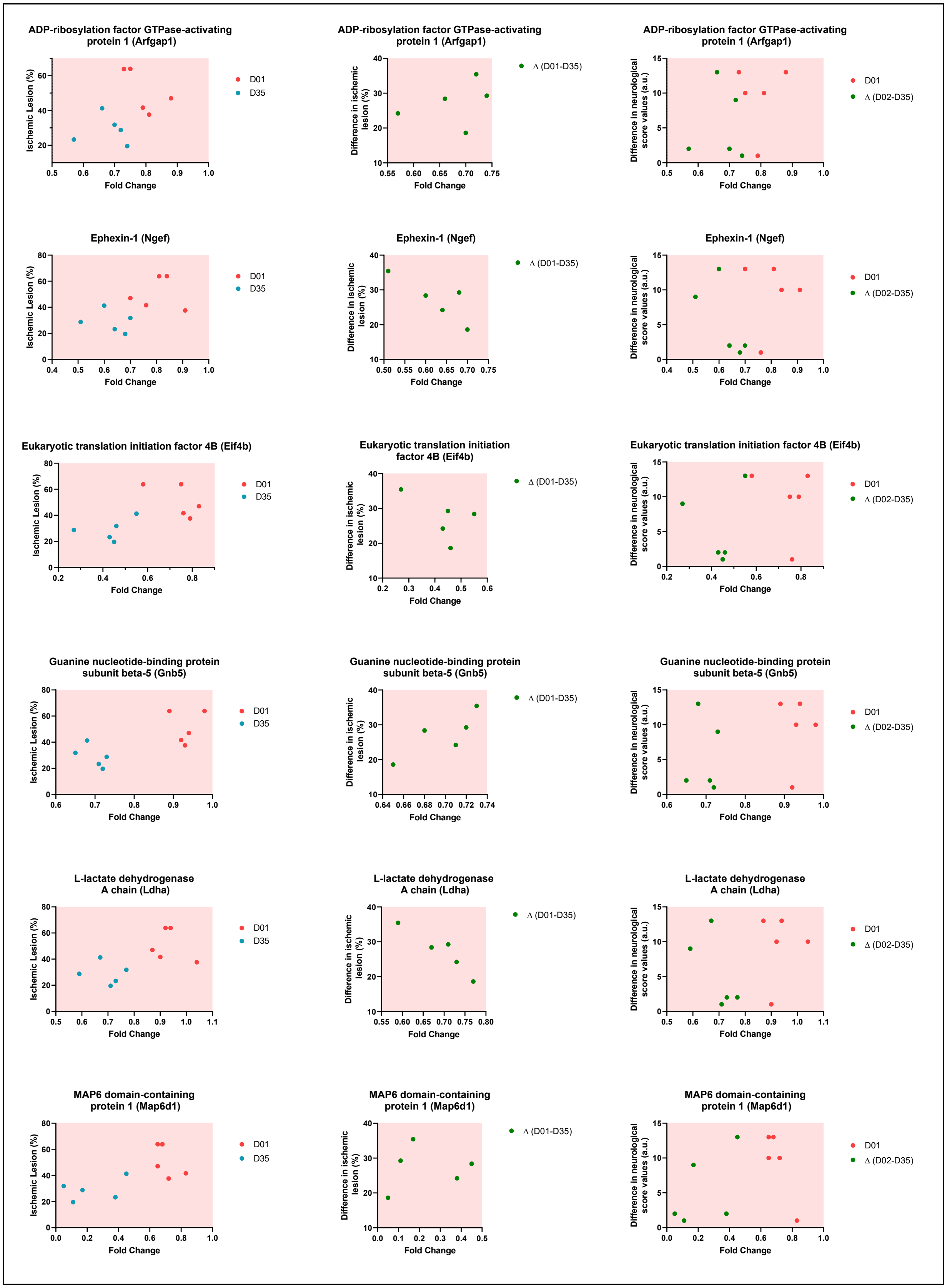

(b2)

acute DOWN_chronic DOWN

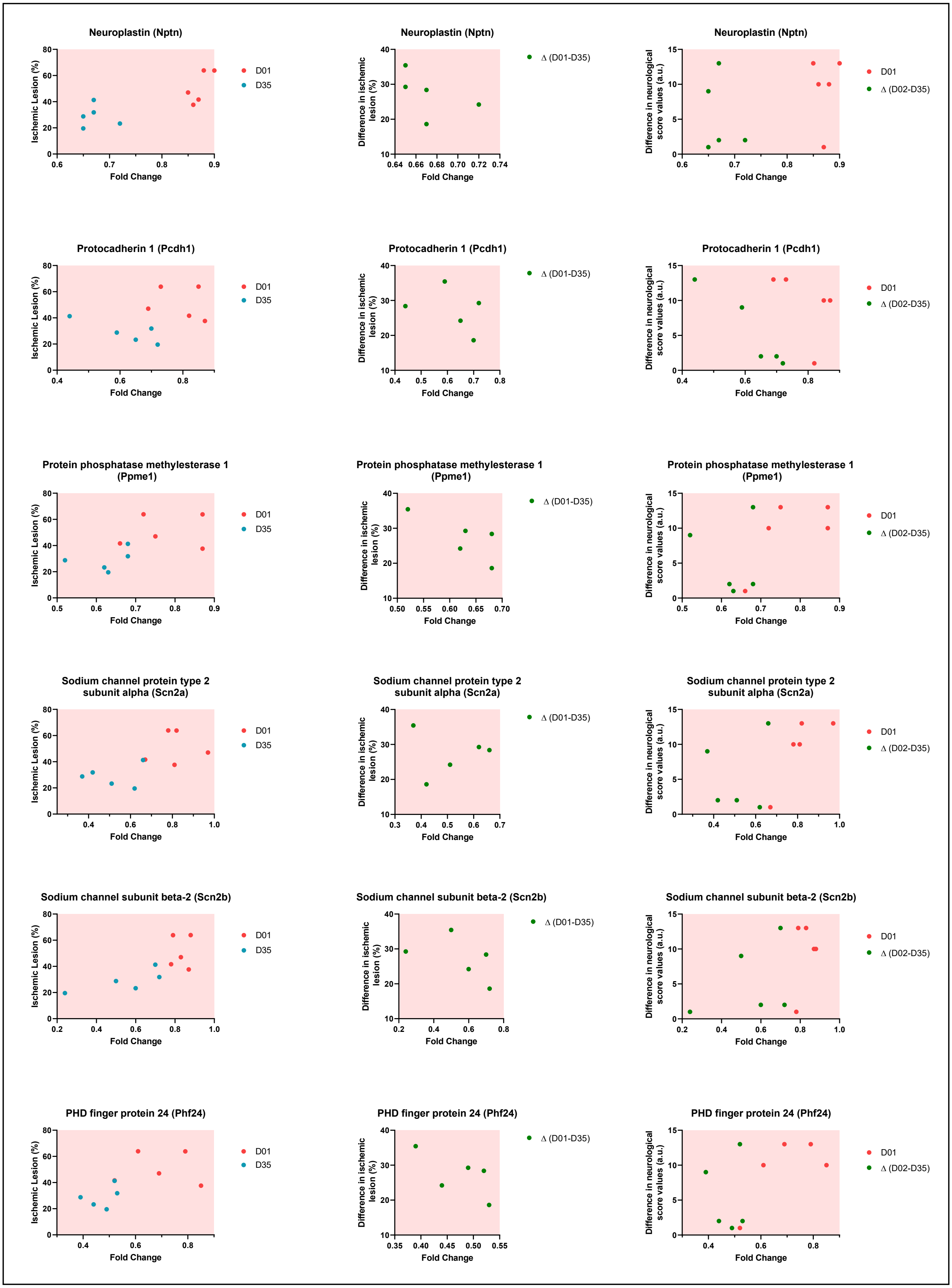

(c)

acute DOWN_chronic UP

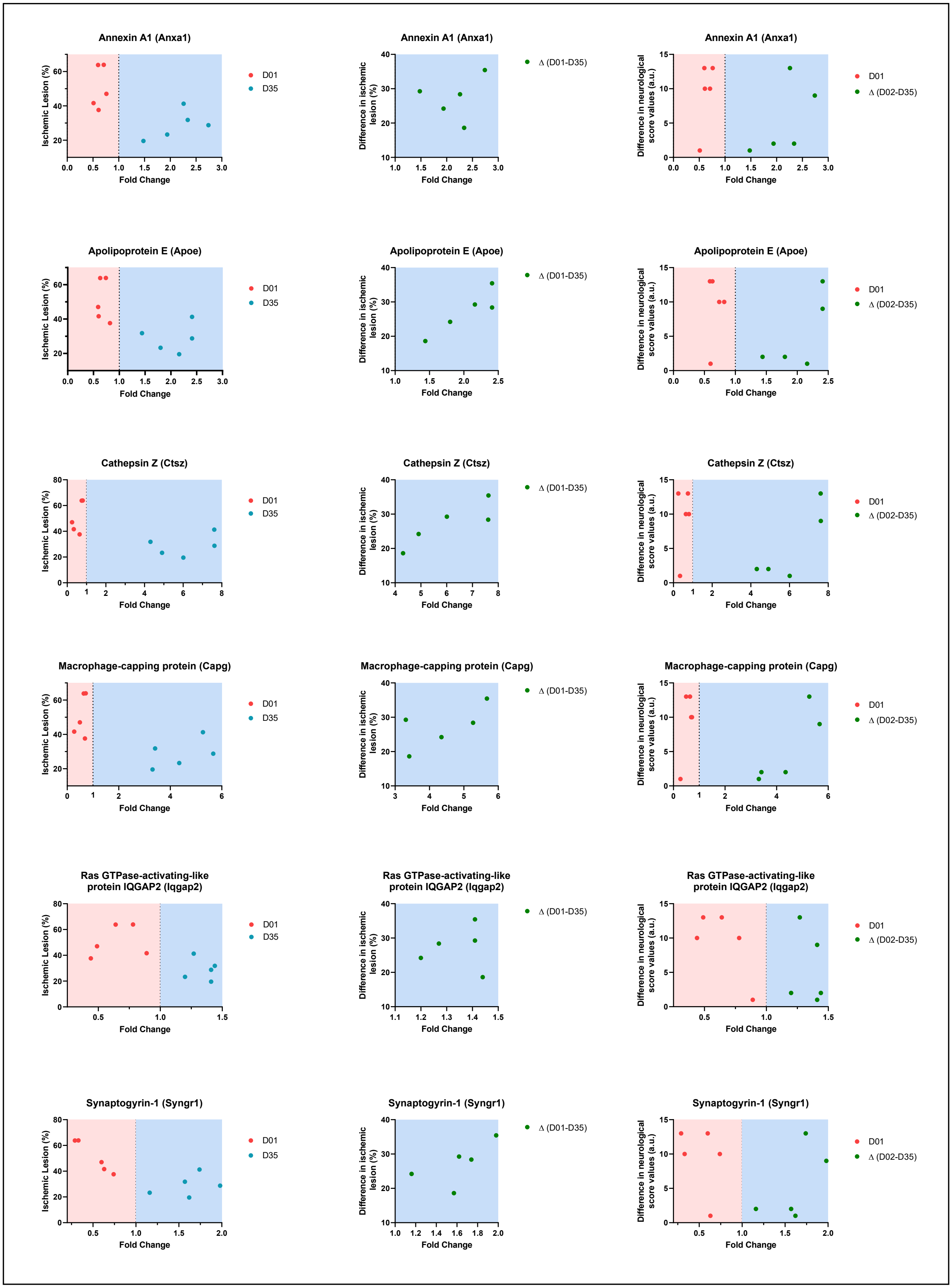

Supplementary Figure 4 Fold change profiles of 46 differentially expressed proteins (DEPs) in the ipsilateral cortex and their association with acute injury and long-term recovery outcomes. Proteins were selected based on the fold change thresholds (FC > 1.3 for upregulation, and < 1.3 for downregulation) and grouped into three temporal expression clusters: **(a1-a6)** acute upregulation with sustained upregulation in the chronic phase (*acute UP_chronic UP*); **(b1-b2)** sustained downregulation (*acute DOWN_chronic DOWN*); **(c)** and acute downregulation with chronic upregulation (*acute DOWN_chronic UP*). Fold change values are shown alongside corresponding measures of: ischemic lesion size in the acute and chronic phases of ischemia; lesion size Δ (D01−D35) representing structural recovery; neurological scores on day 1 (D01 acute deficit); and differences in neurological score values in acute and chronic phase Δ (D02−D35) representing functional recovery

(a1) **acute UP_chronic UP**

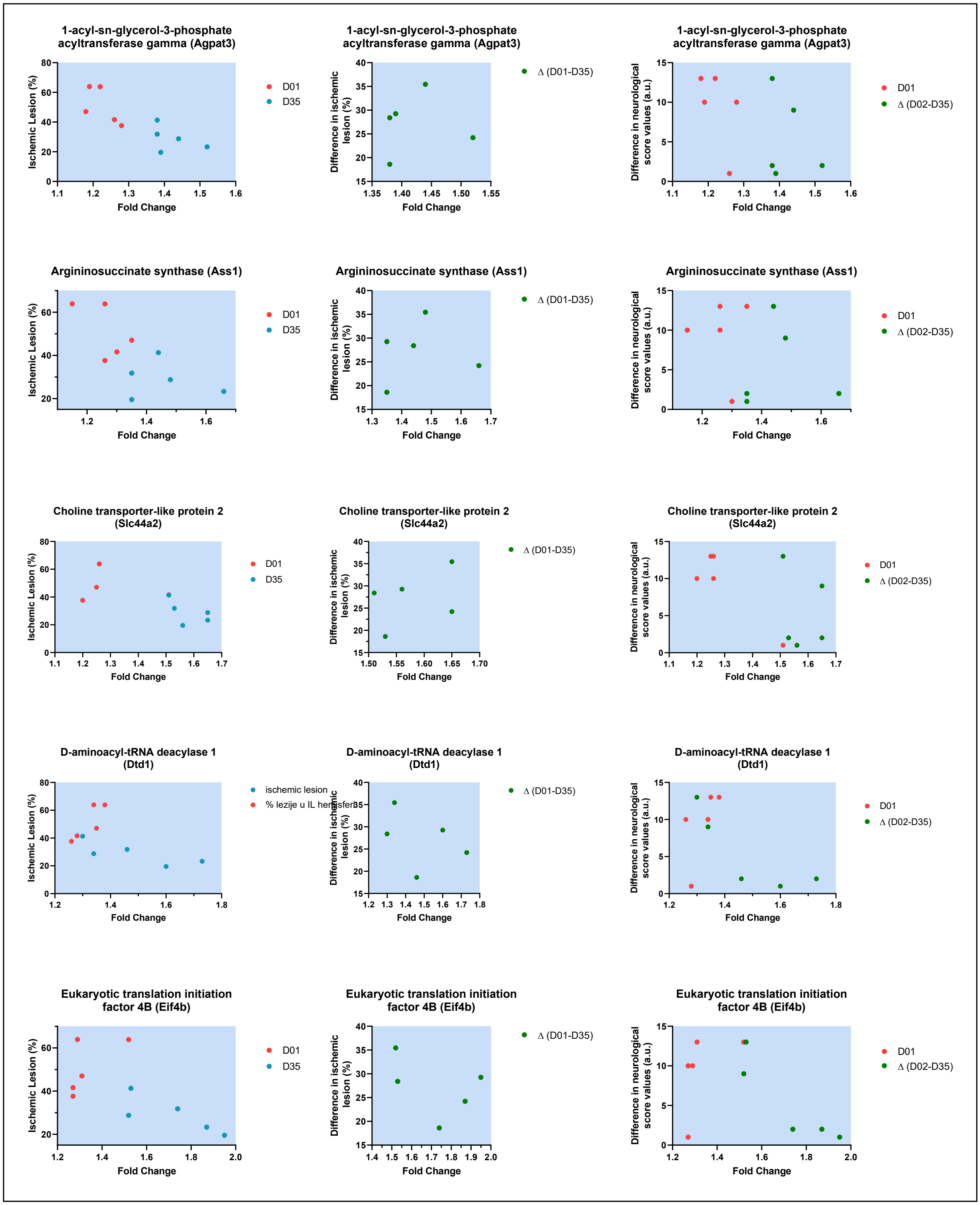

(a2)

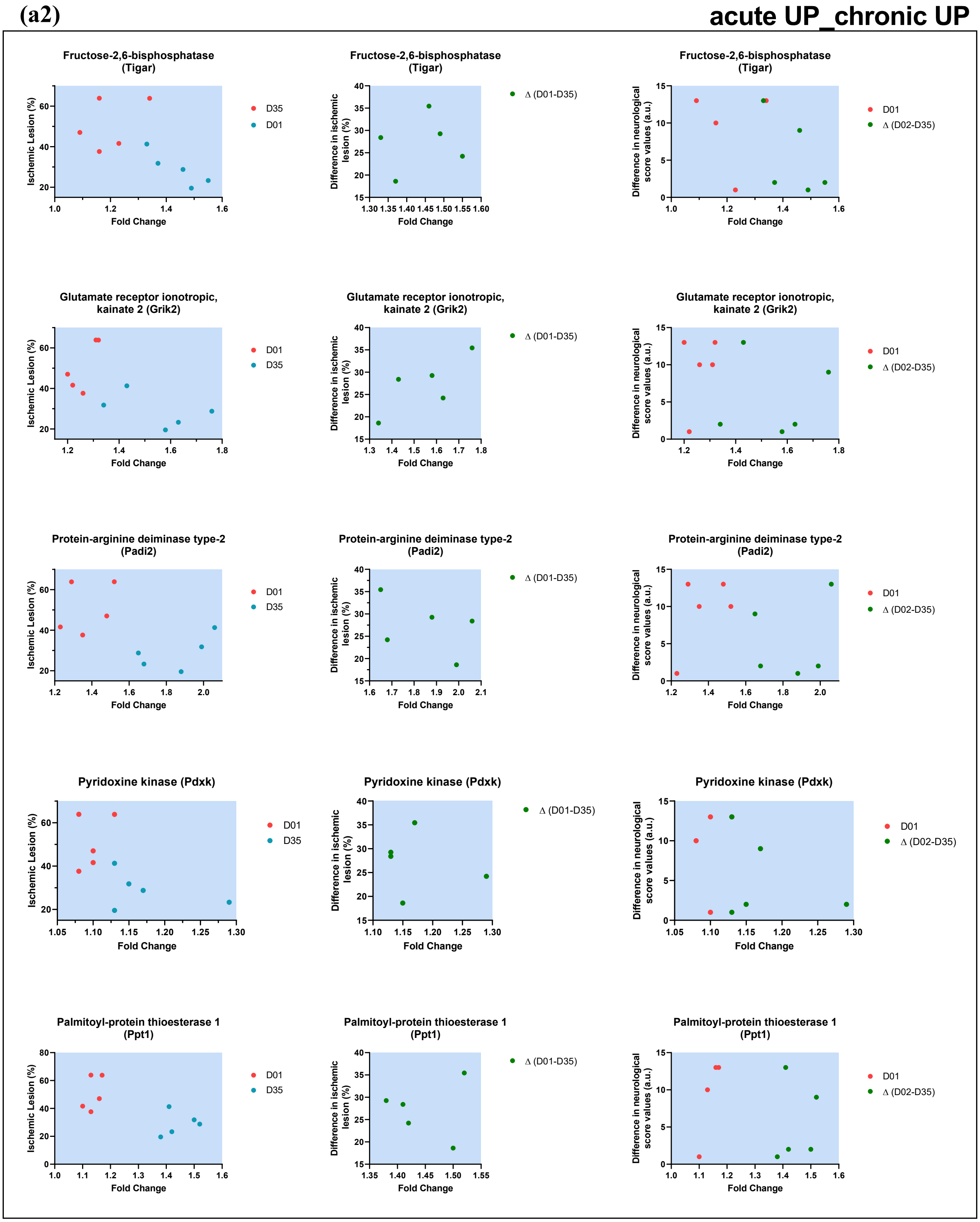
acute UP_chronic UP
Fructose-2,6-bisphosphatase (Tigar)
Glutamate receptor ionotropic, kainate 2 (Grik2)
Protein-arginine deiminase type-2 (Padi2)
Pyridoxine kinase (Pdxk)
Palmitoyl-protein thioesterase 1 (Ppt1)
Ischemic Lesion (%)
Difference in ischemic lesion (%)
Difference in neurological score values (a.u.)
Fold Change
D01
D35
Δ (D01-D35)
Δ (D02-D35)

(a3) **acute UP_chronic UP**

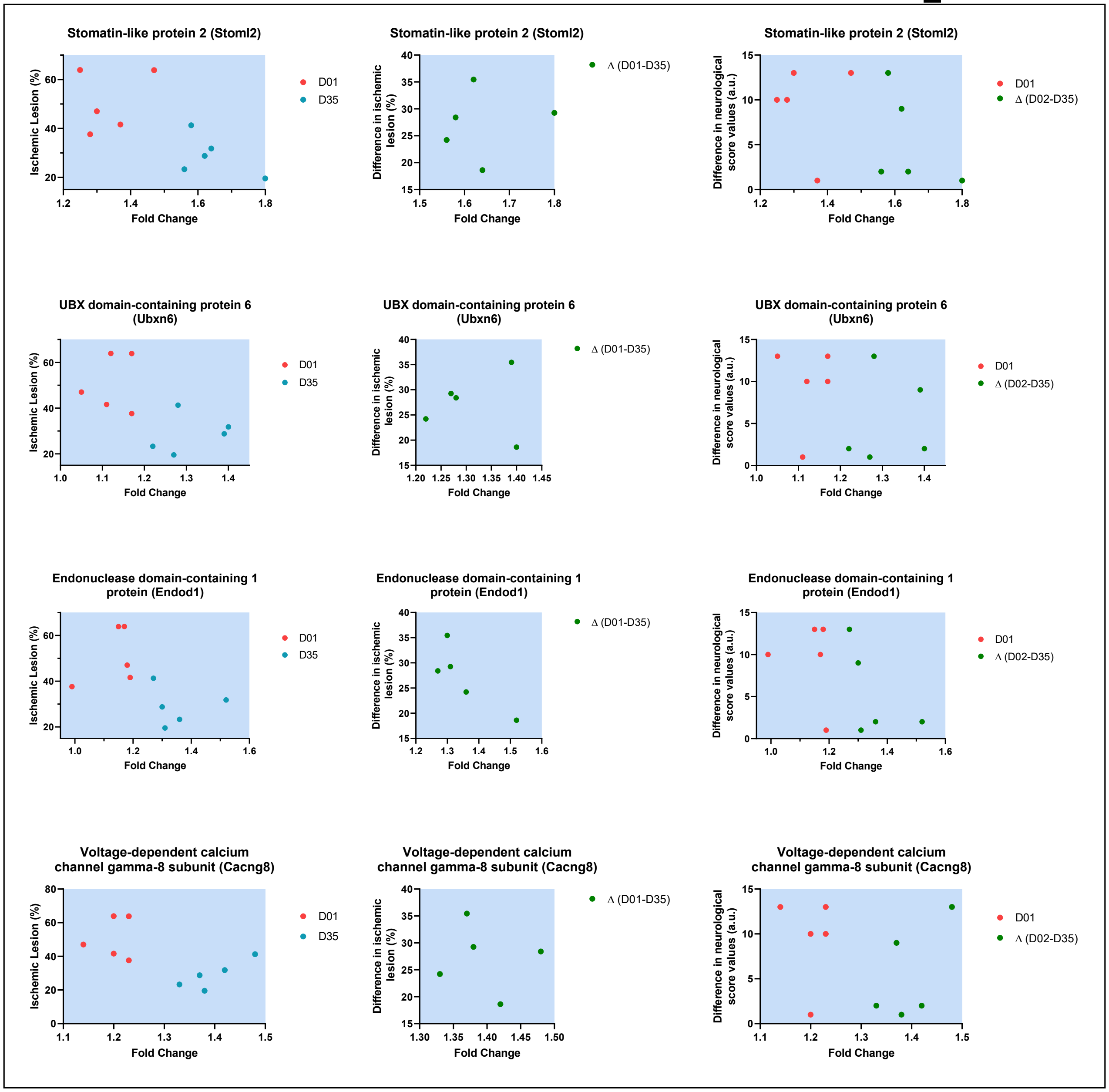

(a4) **acute DOWN_chronic DOWN**

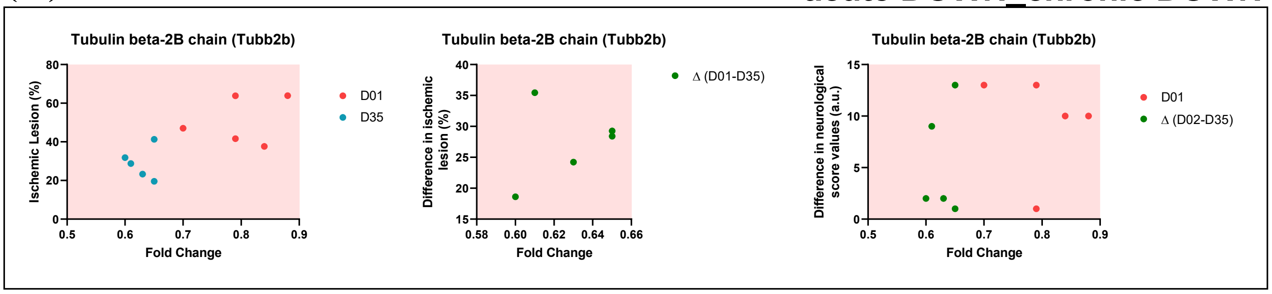

Supplementary Figure 5 Expression profiles of selected proteins in the contralateral cerebral cortex associated with acute ischemic damage and chronic functional and structural recovery. Plots represent fold change values of 15 differentially expressed proteins (DEPs) that were significantly upregulated or downregulated in the acute and/or chronic phases following stroke. Proteins were selected based on the fold change thresholds (FC; > 1.3 for upregulation, and < 1.3 for downregulation) and grouped int two temporal expression clusters: (**a1−a3**) acute upregulation with sustained upregulation in the chronic phase (*acute UP_chronic UP*) and (**a4**) sustained downregulation (*acute DOWN_chronic DOWN*). Fold change values are shown alongside corresponding measures of: ischemic lesion size in the acute and chronic phases of ischemia; lesion size Δ (D01−D35) representing structural recovery; neurological scores on day 1 (D01 acute deficit); and differences in neurological score values in acute and chronic phase Δ (D02−D35) representing functional recovery

1.2 Supplementary Tables

Supplementary Table 1 Imaging parameters and geometric settings for mouse brain *in vivo* MR imaging

MRI SEQUENCE	IMAGING PARAMETERS	GEOMETRICAL PARAMETERS
1. Wobble_position	Repetition time: 500 ms<br>Number of signal averaging: 1	Image size: 128<br>Field of view size: 87 mm
2. Localizer_multi_slice	Echo time: 4 ms<br>Repetition time: 15 ms<br>Number of signal averaging: 1<br>Echo spacing: /	Image orientation: coronal, sagittal and axial<br>Number of images: 3 × 7<br>Image thickness: 0.8 mm<br>Resolution: 0.133 × 0.133 mm
3. T2w_cor_Brain	Echo time: 33 ms<br>Repetition time: 3000 ms<br>Number of signal averaging: 8<br>Echo spacing: 8.25 ms	Image orientation: axial<br>Number of images: 25<br>Image thickness: 0.4 mm<br>Resolution: 0.100 × 0.100 mm
T2map_cor_Brain	Echo time: 7.5 ms<br>Repetition time: 3150 ms<br>Number of signal averaging: 3<br>Echo spacing: 7.5 ms	Image orientation: axial<br>Number of images: 25<br>Image thickness: 0.4 mm<br>Resolution: 0.125 × 0.125 mm

Supplementary Table 2 Calculated p-values for DEPs (ipsilateral and contralateral cortex) based on performed permutation test with *post hoc* Bonferroni correction between three time points

Ipsilateral mouse cortex			*p-values*		
	Accession_No	***Protein Name (Gene name)***	***BL vs D01***	***D01 vs D35***	***BL vs D35***
1.	A0A087WSJ9	Regulator of G-protein signaling 6 (*Rgs6*)	0.00834	0.0156	0.00868
2.	A0A0R4J079	Golgi resident protein GCP60 (*Acbd3*)	0.0482	0.00868	0.00756
3.	A0A0R4J1E2	Elongation factor 1-delta (*Eef1d*)	0.00822	0.00802	0.00776
4.	A0A1B0GR11	Transaldolase (*Taldo1*)	0.00804	0.01558	0.00818
5.	A0A1B0GSX0	L-lactate dehydrogenase A chain (*Ldha*)	0.0707	0.00836	0.00796
6.	A0A1L1STE6	Isocitrate dehydrogenase [NAD] subunit alpha, mitochondrial (*Idh3a*)	0.00754	0.0076	0.0085
7.	A0A5H1ZRM8	Sodium channel protein type 2 subunit alpha (*Scn2a*)	0.02396	0.00786	0.0074
8.	E9PYT3	Atlastin-3 (*Atl3*)	0.0314	0.00752	0.00764
9.	E9Q174	Unconventional myosin-VI (*Myo6*)	0.05608	0.00848	0.00796
10.	E9Q3L2	Phosphatidylinositol 4-kinase alpha (*Pi4ka*)	0.00842	0.00802	0.00794
11.	E9QQ05	Clathrin coat assembly protein AP180 (*Snap91*)	0.01538	0.00734	0.00846
12.	O08529	Calpain-2 catalytic subunit (*Capn2*)	0.01612	0.00868	0.00738
13.	O08532-5	Voltage-dependent calcium channel subunit alpha-2_delta-1 (*Cacna2d1*)	0.04542	0.00744	0.00794
14.	O55026	Ectonucleoside triphosphate diphosphohydrolase 2 (*Entpd2*)	0.08864	0.00812	0.00826
15.	O55100	Synaptogyrin-1 (*Syngr1*)	0.00806	0.00782	0.0077
16.	P00920	Carbonic anhydrase 2 (*Ca2*)	0.02308	0.00778	0.00778
17.	P01027	Complement C3 (*C3*)	0.00824	0.00838	0.00792
18.	P03995-2	Glial fibrillary acidic protein (*Gfap*)	0.02356	0.0076	0.00812
19.	P08226	Apolipoprotein E (*Apoe*)	0.02392	0.00718	0.00804
20.	P08228	Superoxide dismutase [Cu-Zn] (*Sod1*)	0.00774	0.00792	0.00812
21.	P10107	Annexin A1 (*Anxa1*)	0.01618	0.00792	0.0083
22.	P10126	Elongation factor 1-alpha 1 (*Eef1a1*)	0.0321	0.00784	0.00784
23.	P10922	Histone H1.0 (*H1-0*)	0.00822	0.00858	0.00854
24.	P11983	T-complex protein 1 subunit alpha (*Tcp1*)	0.008	0.01596	0.00766
25.	P16460	Argininosuccinate synthase (*Ass1*)	0.00786	0.00742	0.00748
26.	P18242	Cathepsin D (*Ctsd*)	0.03208	0.00758	0.00712
27.	P19157	Glutathione S-transferase P 1 (*Gstp1*)	0.00752	0.0074	0.00672
28.	P23116	Eukaryotic translation initiation factor 3 subunit A (*Eif3a*)	0.02422	0.0075	0.00802
29.	P31786	Acyl-CoA-binding protein (*Dbi*)	0.04602	0.00862	0.0078
30.	P31938	Dual specificity mitogen-activated protein kinase kinase 1 (*Map2k1*)	0.00896	0.00846	0.00834
31.	P32037	Solute carrier family 2, facilitated glucose transporter member 3 (*Slc2a3*)	0.00832	0.00796	0.00842
32.	P40237	CD82 antigen (*Cd82*)	0.02332	0.0078	0.00838

33.	P47857-3	ATP-dependent 6-phosphofructokinase, muscle type (*Pfkm*)	0.01568	0.00758	0.01606
34.	P62774	Myotrophin (*Mtpn*)	0.00798	0.00832	0.02456
35.	P62806	Histone H4 (*H4c16*)	0.03244	0.00836	0.0086
36.	P62881-2	Guanine nucleotide-binding protein subunit beta-5 (*Gnb5*)	0.04038	0.008	0.00802
37.	P62962	Profilin-1 (*Pfn1*)	0.02444	0.00772	0.00792
38.	P80315	T-complex protein 1 subunit delta (*Cct4*)	0.00758	0.05486	0.00752
39.	P97300	Neuroplastin (*Nptn*)	0.03136	0.00762	0.00722
40.	P97797-2	Tyrosine-protein phosphatase non-receptor type substrate 1 (*Sirpa*)	0.00852	0.0075	0.00808
41.	Q06890	Clusterin (*Clu*)	0.0235	0.00826	0.008
42.	Q14BB9	MAP6 domain-containing protein 1 (*Map6d1*)	0.00812	0.00792	0.0077
43.	Q3TCR7	Dynamin-2 (*Dnm2*)	0.01626	0.00774	0.0076
44.	Q3TKX1	V-type proton ATPase subunit S1 (*Atp6ap1*)	0.0075	0.00816	0.00856
45.	Q3UQ44	Ras GTPase-activating-like protein IQGAP2 (*Iqgap2*)	0.0155	0.00852	0.0086
46.	Q3UY21	Myelin-oligodendrocyte glycoprotein (*Mog*)	0.0245	0.00876	0.00782
47.	Q543K9	Purine nucleoside phosphorylase (*Pnp*)	0.07102	0.00762	0.00804
48.	Q56A07	Sodium channel subunit beta-2 (*Scn2b*)	0.00762	0.00838	0.00804
49.	Q61425	Hydroxyacyl-coenzyme A dehydrogenase, mitochondrial (*Hadh*)	0.01572	0.00716	0.00756
50.	Q6NS82	Reticulophagy regulator 2 (*Retreg2*)	0.00784	0.00802	0.02412
51.	Q6P1J1	Dihydropyrimidinase-related protein 1 (*Crmp1*)	0.03958	0.00796	0.00882
52.	Q78PY7	Staphylococcal nuclease domain-containing protein 1 (*Snd1*)	0.04828	0.00768	0.00864
53.	Q80TL4	PHD finger protein 24 (*Phf24*)	0.00764	0.01578	0.00794
54.	Q80UE5	Band 4.1-like protein 2 (*Epb41l2*)	0.04702	0.00788	0.0084
55.	Q8BG33	Neurotrimin (*Ntm*)	0.0082	0.00796	0.00788
56.	Q8BGD9	Eukaryotic translation initiation factor 4B (*Eif4b*)	0.0312	0.00834	0.00764
57.	Q8BT60	Copine-3 (*Cpne3*)	0.02466	0.00704	0.00834
58.	Q8BVI4	Dihydropteridine reductase (*Qdpr*)	0.00788	0.00824	0.0085
59.	Q8BVQ5	Protein phosphatase methylesterase 1 (*Ppme1*)	0.00756	0.02488	0.00808
60.	Q8CFX3	Protocadherin 1 (*Pcdh1*)	0.00806	0.02426	0.0078
61.	Q8CHT1	Ephexin-1 (*Ngef*)	0.00824	0.0156	0.00794
62.	Q8VDN2	Sodium_potassium-transporting ATPase subunit alpha-1 (*Atp1a1*)	0.00802	0.00772	0.00802
63.	Q91V64	Isochorismatase domain-containing protein 1 (*Isoc1*)	0.03266	0.00856	0.0075
64.	Q99LB4	Macrophage-capping protein (*Capg*)	0.00788	0.008	0.00788
65.	Q9CQ60	6-phosphogluconolactonase (*Pgls*)	0.02408	0.00768	0.00822
66.	Q9CQQ7	ATP synthase F(0) complex subunit B1, mitochondrial (*Atp5pb*)	0.00806	0.00844	0.0087
67.	Q9D554	Splicing factor 3A subunit 3 (*Sf3a3*)	0.00804	0.00788	0.00798
68.	Q9DAR7	m7GpppX diphosphatase (*Dcps*)	0.02348	0.0155	0.00736
69.	Q9DBJ1	Phosphoglycerate mutase 1 (*Pgam1*)	0.00746	0.00752	0.02322
70.	Q9WUU7	Cathepsin Z (*Ctsz*)	0.00754	0.00806	0.0075
71.	Q9WV34-2	MAGUK p55 subfamily member 2 (*Mpp2*)	0.0081	0.00806	0.00702
72.	Q9Z0P5	Twinfilin-2 (*Twf2*)	0.00778	0.0077	0.00784
73.	V9GX26	Glycogenin-1 (*Gyg1*)	0.02294	0.00814	0.00818

74.	V9GXM1	ADP-ribosylation factor GTPase-activating protein 1 (*Arfgap1*)	0.00762	0.01572	0.00746
**Contralateral mouse cortex**			***p-values***		
	Accession_No	***Protein Name (Gene name)***	***BL vs D01***	***D01 vs D35***	***BL vs D35***
1.	Q8K183	Pyridoxine kinase (*Pdxk*)	0.00812	0.00866	0.00742
2.	P16460	Argininosuccinate synthase (*Ass1*)	0.01432	0.00746	0.00792
3.	Q8BGD9	Eukaryotic translation initiation factor 4B (*Eif4b*)	0.0077	0.0162	0.008
4.	Q99PL6	UBX domain-containing protein 6 (*Ubxn6*)	0.01618	0.00794	0.00798
5.	Q08642	Protein-arginine deiminase type-2 (*Padi2*)	0.01566	0.00798	0.0083
6.	Q99JB2	Stomatin-like protein 2 (*Stoml2*)	0.00864	0.00786	0.00764
7.	Q8C522	Endonuclease domain-containing 1 protein (*Endod1*)	0.03968	0.00786	0.0074
8.	Q8BY89	Choline transporter-like protein 2 (*Slc44a2*)	0.01644	0.01536	0.0083
9.	O88531	Palmitoyl-protein thioesterase 1 (*Ppt1*)	0.00784	0.0083	0.00762
10.	Q9DD18	D-aminoacyl-tRNA deacylase 1 (*Dtd1*)	0.00766	0.09522	0.0085
11.	Q9D517	1-acyl-sn-glycerol-3-phosphate acyltransferase gamma (*Agpat3*)	0.00818	0.0075	0.00814
12.	Q8BZA9	Fructose-2,6-bisphosphatase (*Tigar*)	0.03264	0.01522	0.00786
13.	Q8VHW2	Voltage-dependent calcium channel gamma-8 subunit (*Cacng8*)	0.01546	0.0076	0.00708
14.	P39087	Glutamate receptor ionotropic, kainate 2 (*Grik2*)	0.00712	0.00826	0.00812
15.	Q9CWF2	Tubulin beta-2B chain (*Tubb2b*)	0.00826	0.00768	0.00792

Supplementary Table 3 List of 74 differently expressed proteins (DEPs) from ipsilateral cortex and their respective fold change (FC) values for acute and chronic phases. Cells with FC values higher than 1.3 ($p < 0.05$) are highlighted green, and lower than 0.7 ($p < 0.05$) red

No.	Acession No.	Protein Name (Gene Name)	Fold Change	
			CTRL vs D01	CTRL vs D35
1.	A0A0R4J079_MOUSE	Golgi resident protein GCP60 (*Acbd3*)	0.91	1.18
2.	ANXA1_MOUSE	Annexin A1 (*Anxa1*)	0.64	2.15
3.	APOE_MOUSE	Apolipoprotein E (*Apoe*)	0.68	2.04
4.	V9GXM1_MOUSE	ADP-ribosylation factor GTPase-activating protein 1 (*Arfgap1*)	0.79	0.68
5.	ASSY_MOUSE	Argininosuccinate synthase (*Ass1*)	1.24	1.78
6.	E9PYT3_MOUSE	Atlastin-3 (*Atl3*)	1.29	1.81
7.	AT1A1_MOUSE	Sodium_potassium-transporting ATPase subunit alpha-1 (*Atp1a1*)	0.92	0.74
8.	AT5F1_MOUSE	ATP synthase F(0) complex subunit B1, mitochondrial (*Atp5pb*)	1.13	0.89
9.	Q3TKX1_MOUSE	V-type proton ATPase subunit S1 (*Atp6ap1*)	1.14	0.83
10.	CO3_MOUSE	Complement C3 (*C3*)	4.19	11.79
11.	CAH2_MOUSE	Carbonic anhydrase 2 (*Ca2*)	1.21	1.73
12.	CA2D1_MOUSE	Voltage-dependent calcium channel subunit alpha-2_delta-1 (*Cacna2d1*)	0.91	0.70
13.	Q99LB4_MOUSE	Macrophage-capping protein (*Capg*)	0.56	4.40
14.	CAN2_MOUSE	Calpain-2 catalytic subunit (*Capn2*)	0.93	1.22
15.	TCPD_MOUSE	T-complex protein 1 subunit delta (*Cct4*)	1.13	1.18
16.	CD82_MOUSE	CD82 antigen (*Cd82*)	1.16	1.50
17.	CLUS_MOUSE	Clusterin (*Clu*)	1.44	3.80
18.	CPNE3_MOUSE	Copine-3 (*Cpne3*)	1.13	1.94
19.	Q6P1J1_MOUSE	Dihydropyrimidinase-related protein 1 (*Crmp1*)	0.94	0.79
20.	CATD_MOUSE	Cathepsin D (*Ctsd*)	0.89	4.31
21.	CATZ_MOUSE	Cathepsin Z (*Ctsz*)	0.55	6.09
22.	ACBP_MOUSE	Acyl-CoA-binding protein (*Dbi*)	1.15	2.48
23.	DCPS_MOUSE	m7GpppX diphosphatase (*Dcps*)	1.27	1.62
24.	Q3TCR7_MOUSE	Dynamin-2 (*Dnm2*)	1.39	2.13
25.	EF1A1_MOUSE	Elongation factor 1-alpha 1 (*Eef1a1*)	1.13	1.50
26.	A0A0R4J1E2_MOUSE	Elongation factor 1-delta (*Eef1d*)	0.90	1.16
27.	EIF3A_MOUSE	Eukaryotic translation initiation factor 3 subunit A (*Eif3a*)	1.11	1.27
28.	IF4B_MOUSE	Eukaryotic translation initiation factor 4B (*Eif4b*)	0.74	0.43
29.	ENTP2_MOUSE	Ectonucleoside triphosphate diphosphohydrolase 2 (*Entpd2*)	1.19	1.78
30.	Q80UE5_MOUSE	Band 4.1-like protein 2 (*Epb41l2*)	0.90	1.21
31.	GFAP_MOUSE	Glial fibrillary acidic protein (*Gfap*)	1.34	9.80
32.	GNB5_MOUSE	Guanine nucleotide-binding protein subunit beta-5 (*Gnb5*)	0.93	0.70
33.	GSTP1_MOUSE	Glutathione S-transferase P 1 (*Gstp1*)	1.12	1.44
34.	V9GX26_MOUSE	Glycogenin-1 (*Gyg1*)	1.41	2.07
35.	H10_MOUSE	Histone H1.0 (*H1-0*)	1.27	1.95

36.	H4_MOUSE	Histone H4 (*H4c16*)	1.13	1.77
37.	HCDH_MOUSE	Hydroxyacyl-coenzyme A dehydrogenase, mitochondrial (*Hadh*)	1.18	1.66
38.	A0A1L1STE6_MOUSE	Isocitrate dehydrogenase [NAD] subunit alpha, mitochondrial (*Idh3a*)	1.10	0.93
39.	IQGA2_MOUSE	Ras GTPase-activating-like protein IQGAP2 (*Iqgap2*)	0.65	1.35
40.	ISOC1_MOUSE	Isochorismatase domain-containing protein 1 (*Isoc1*)	1.37	1.84
41.	A0A1B0GSX0_MOUSE	L-lactate dehydrogenase A chain (*Ldha*)	0.93	0.70
42.	MP2K1_MOUSE	Dual specificity mitogen-activated protein kinase kinase 1 (*Map2k1*)	0.91	0.82
43.	MA6D1_MOUSE	MAP6 domain-containing protein 1 (*Map6d1*)	0.71	0.23
44.	Q3UY21_MOUSE	Myelin-oligodendrocyte glycoprotein (*Mog*)	1.25	1.67
45.	MPP2_MOUSE	MAGUK p55 subfamily member 2 (*Mpp2*)	0.94	0.80
46.	MTPN_MOUSE	Myotrophin (*Mtpn*)	0.86	1.08
47.	E9Q174_MOUSE	Unconventional myosin-VI (*Myo6*)	0.92	1.44
48.	NGEF_MOUSE	Ephexin-1 (*Ngef*)	0.80	0.63
49.	NPTN_MOUSE	Neuroplastin (*Nptn*)	0.87	0.67
50.	Q8BG33_MOUSE	Neurotrimin (*Ntm*)	0.92	0.79
51.	Q8CFX3_MOUSE	Protocadherin 1 (*Pcdh1*)	0.79	0.62
52.	PFKAM_MOUSE	ATP-dependent 6-phosphofructokinase, muscle type (*Pfkm*)	1.04	0.90
53.	PROF1_MOUSE	Profilin-1 (*Pfn1*)	1.09	1.41
54.	PGAM1_MOUSE	Phosphoglycerate mutase 1 (*Pgam1*)	1.07	0.94
55.	6PGL_MOUSE	6-phosphogluconolactonase (*Pgls*)	1.21	1.54
56.	PHF24_MOUSE	PHD finger protein 24 (*Phf24*)	0.69	0.47
57.	PI4KA_MOUSE	Phosphatidylinositol 4-kinase alpha (*Pi4ka*)	1.08	0.84
58.	Q543K9_MOUSE	Purine nucleoside phosphorylase (*Pnp*)	1.11	1.95
59.	PPME1_MOUSE	Protein phosphatase methylesterase 1 (*Ppme1*)	0.77	0.63
60.	DHPR_MOUSE	Dihydropteridine reductase (*Qdpr*)	1.28	1.66
61.	RETR2_MOUSE	Reticulophagy regulator 2 (*Retreg2*)	1.21	0.85
62.	A0A087WSJ9_MOUSE	Regulator of G-protein signaling 6 (*Rgs6*)	0.85	0.72
63.	A0A5H1ZRM8_MOUSE	Sodium channel protein type 2 subunit alpha (*Scn2a*)	0.81	0.51
64.	SCN2B_MOUSE	Sodium channel subunit beta-2 (*Scn2b*)	0.83	0.55
65.	SF3A3_MOUSE	Splicing factor 3A subunit 3 (*Sf3a3*)	1.35	2.10
66.	SHPS1_MOUSE	Tyrosine-protein phosphatase non-receptor type substrate 1 (*Sirpa*)	0.88	0.72
67.	GTR3_MOUSE	Solute carrier family 2, facilitated glucose transporter member 3 (*Slc2a3*)	1.15	0.86
68.	E9QQ05_MOUSE	Clathrin coat assembly protein AP180 (*Snap91*)	0.93	0.74
69.	SND1_MOUSE	Staphylococcal nuclease domain-containing protein 1 (*Snd1*)	0.94	1.16

70.	SODC_MOUSE	Superoxide dismutase [Cu-Zn] (*Sod1*)	0.88	1.19
71.	SNG1_MOUSE	Synaptogyrin-1 (*Syngr1*)	0.52	1.61
72.	A0A1B0GR11_MOUSE	Transaldolase (*Taldo1*)	1.35	1.58
73.	TCPA_MOUSE	T-complex protein 1 subunit alpha (*Tcp1*)	1.13	1.18
74.	TWF2_MOUSE	Twinfilin-2 (*Twf2*)	0.81	0.89

Supplementary Table 4 List of 15 differently expressed proteins (DEPs) from the contralateral cortex and their respective fold change (FC) values for acute and chronic phases. Cells with FC values higher than 1.3 ($p < 0.05$) are highlighted green, and lower than 0.7 ($p < 0.05$) red

No.	Acession No.	Protein Name (Gene Name)	Fold Change	
			CTRL vs D01	CTRL vs D35
1.	PDXK_MOUSE	Pyridoxine kinase (*Pdxk*)	1.10	1.17
2.	ASSY_MOUSE	Argininosuccinate synthase (*Ass1*)	1.26	1.46
3.	IF4B_MOUSE	Eukaryotic translation initiation factor 4B (*Eif4b*)	1.33	1.72
4.	UBXN6_MOUSE	UBX domain-containing protein 6 (*Ubxn6*)	1.13	1.31
5.	PADI2_MOUSE	Protein-arginine deiminase type-2 (*Padi2*)	1.37	1.85
6.	STML2_MOUSE	Stomatin-like protein 2 (*Stoml2*)	1.33	1.64
7.	ENDD1_MOUSE	Endonuclease domain-containing 1 protein (*Endod1*)	1.14	1.35
8.	CTL2_MOUSE	Choline transporter-like protein 2 (*Slc44a2*)	1.29	1.58
9.	PPT1_MOUSE	Palmitoyl-protein thioesterase 1 (*Ppt1*)	1.14	1.45
10.	DTD1_MOUSE	D-aminoacyl-tRNA deacylase 1 (*Dtd1*)	1.32	1.49
11.	PLCC_MOUSE	1-acyl-sn-glycerol-3-phosphate acyltransferase gamma (*Agpat3*)	1.23	1.42
12.	TIGAR_MOUSE	Fructose-2,6-bisphosphatase (*Tigar*)	1.20	1.44
13.	CCG8_MOUSE	Voltage-dependent calcium channel gamma-8 subunit (*Cacng8*)	1.20	1.40
14.	GRIK2_MOUSE	Glutamate receptor ionotropic, kainate 2 (*Grik2*)	1.26	1.55
15.	TBB2B_MOUSE	Tubulin beta-2B chain (*Tubb2b*)	0.80	0.63
